# Copper(I)-Anchoring Covalent Organic Polymer for Heterogeneous CuAAC Reaction without Reducing Agents and Copper Leaching

**DOI:** 10.1021/acsorginorgau.5c00067

**Published:** 2025-09-25

**Authors:** Maria Aurora Guarducci, Simone Manetto, Andrea Giacomo Marrani, Francesco Amato, Paolo Guglielmi, Michele Coluccia, Antonella Fontana, Serena Pilato, Claudio Villani, Alessia Ciogli, Giulia Mazzoccanti

**Affiliations:** † Department of Chemistry and Technologies of Drugs, 9311 Sapienza University of Rome, pl.e A. Moro 5, 00185 Roma, Italy; ‡ Department of Chemistry, Sapienza University of Rome, pl.e A. Moro 5, 00185 Roma, Italy; § UdA-Tech Lab Research Center, “G. d’Annunzio” University of Chieti-Pescara, 66100 Chieti, Italy; ∥ Department of Pharmacy, “G. d’Annunzio” University of Chieti-Pescara, 66100 Chieti, Italy

**Keywords:** heterogeneous catalysis, CuAAC, covalent organic polymer, copper(I), metal leaching prevention

## Abstract

A novel copper­(I)-anchored covalent organic polymer (Cu^+^@COP) is presented as a robust, heterogeneous catalyst for copper-catalyzed azide–alkyne cycloaddition (CuAAC), operating without the need for external reducing agents or observable copper leaching. Cu^+^ stabilization is achieved via multidentate N,O-ligand coordination within the polymer matrix, enabling high catalytic efficiency (up to 95% yield) and recyclability. Structural, spectroscopic, and ICP-OES analyses confirm Cu presence, offering an alternative to traditional CuAAC protocols. This system combines operational simplicity, reduced waste, and green chemistry principles, positioning Cu^+^@COP as a practical catalyst for applications in synthetic and materials chemistry.

## Graphical abstract

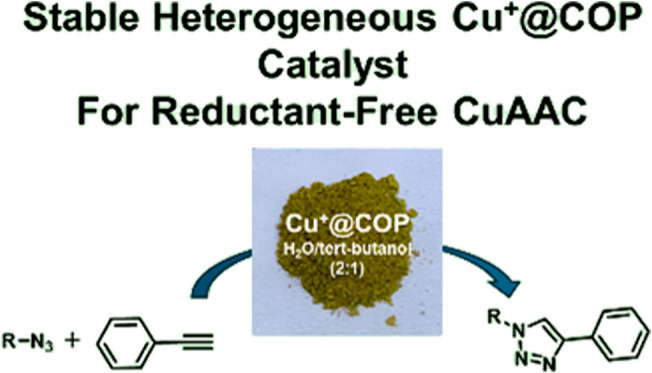

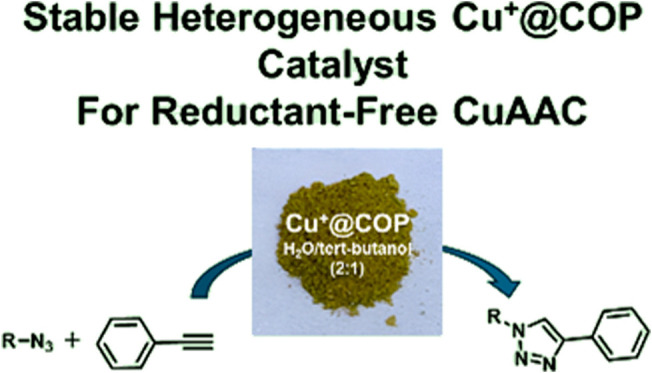

## 1 Introduction

Click chemistry has revolutionized synthetic methodologies by enabling efficient and selective transformations for constructing complex molecular architectures. The fundamental importance of this approach was recognized with the Nobel Prize in Chemistry 2022, awarded to K. Barry Sharpless, Morten Meldal, and Carolyn R. Bertozzi for “the development of click chemistry and bioorthogonal chemistry.” The seminal works by Sharpless and Meldal independently reporting the Cu­(I)-catalyzed azide–alkyne cycloaddition^1,2^ and Bertozzi’s pioneering bioorthogonal chemistry^3^ laid the foundation for this transformative field. Among click chemistry approaches, the copper­(I)-catalyzed azide–alkyne cycloaddition (CuAAC) has become a benchmark reaction due to its regioselectivity, biocompatibility, and broad applicability in materials science, bioconjugation, and pharmaceutical chemistry.^4–6^ The reaction produces stable 1,4-disubstituted triazoles under mild conditions, making it a powerful tool for linking molecular components.^4^ However, a persistent challenge in CuAAC catalysis is the need for continuous reduction of Cu­(II) to Cu­(I) using external reducing agents such as sodium ascorbate, which can introduce side reactions, catalyst deactivation, and complex purification steps.^7^ Additionally, concerns over copper toxicity and leaching pose significant challenges, particularly in biomedical and pharmaceutical applications.^7^ To mitigate these issues, heterogeneous CuAAC catalysts have been developed, offering advantages such as enhanced stability, recyclability, and easier catalyst recovery.^4^ Several copper-supported systems, including covalent organic frameworks (COFs), metal–organic frameworks (MOFs), and polymer-supported catalysts, have been investigated for CuAAC reactions.^5,8–12^ However, these systems are often limited by structural instability, copper leaching, and reliance on reducing agents to maintain Cu­(I) activity. While some approaches, such as deep eutectic solvents (DESs)^13,14^ and electrochemical stabilization,^15,16^ have been proposed to maintain Cu­(I) without external reductants, no studies have systematically investigated heterogeneous CuAAC catalysis where Cu­(I) stability is ensured without reducing agents. Given that Cu­(I) readily oxidizes to Cu­(II) in air due to its standard reduction potential (+0.16 V vs standard hydrogen electrode SHE in aqueous solution),^17^ this remains a key challenge.

In recent years, several benchmark heterogeneous CuAAC systems have been reported, providing valuable insights into copper immobilization and catalytic efficiency. Neumann et al.^4^ reviewed both homogeneous and heterogeneous CuAAC, highlighting that many polymer-supported systems, while offering recyclability, still require continuous reduction of Cu­(II) to Cu­(I) during catalysis. Binder and co-workers^18^ demonstrated MOF-based systems with high recyclability, yet these relied on porous architectures and often showed gradual Cu leaching. Palmans et al.^19^ reported polymeric Cu catalysts enabling efficient click chemistry not requiring a reductant to maintain Cu­(I) activity. These studies emphasize that while structural design can improve catalyst robustness, complete elimination of reducing agents without loss of performance remains largely unexplored. Other innovative approaches include single-chain nanoparticles (SCNPs) encapsulating Cu species^20,21^ which mimic enzymatic microenvironments to stabilize Cu­(I) and accelerate CuAAC in water. In one of these works, Chen et al.^21^ showed that confined hydrophobic domains protect Cu­(I) from oxidation, while maintaining accessibility for substrates. Previous studies have demonstrated that Cu­(I) complexes with N-donor ligands, such as pyrazoles, phosphines, and pyridine-based systems, exhibit enhanced stability by forming strong coordination bonds that modulate electron density around the metal center and prevent oxidation.^22–27^ In this work, we introduce an insoluble polymer, (here named covalent organic polymer (COP) because it displays chemical stability akin to COFs but lacks long-range crystallinity and porosity), functionalized with Cu­(I) sites, designed to serve as a stable, recyclable heterogeneous CuAAC catalyst. In our system, Cu­(I) works without requiring additional reducing agents. This can be attributed to multidentate ligand coordination, where pyridine and amide functionalities anchor Cu­(I), limit mobility and exposure to oxidative species. Additionally, the polymeric structure provides steric protection, reducing oxygen accessibility and further preventing oxidation.^22^ This combination of strong ligand coordination, steric hindrance, and polymeric immobilization enables Cu­(I) stabilization under aqueous conditions, ensuring efficient and sustainable CuAAC catalysis. Thus, unlike COFs and MOFs, this system does not rely on porosity but instead takes advantage of anchoring of copper species within a robust polymer matrix.^4,28^ The polymer was synthesized via direct amidation between pyridine-2,6-dicarboxylic acid and tetrakis­(4-aminophenyl)­methane, followed by Cu­(II) coordination and in situ reduction to generate Cu^+^@COP. Notably, this catalyst performs CuAAC reactions without requiring additional reducing agents, simplifying reaction conditions and enhancing operational stability. Crucially, Inductively Coupled Plasma Optical Emission Spectroscopy (ICP-OES) analysis confirmed that the copper content remains unchanged before and after catalysis, demonstrating no detectable metal leaching. X-ray Photoelectron Spectroscopy (XPS) analysis further revealed that both Cu­(I) and Cu­(II) species coexist in Cu^+^@COP, indicating that some Cu­(II) persists after reduction. Despite this, the catalyst exhibits high efficiency in CuAAC reactions, highlighting its stability and suitability for applications where strict control over metal residues is essential. By eliminating the need for reducing agents and preventing metal contamination, this system simplifies purification, improves sustainability, and enhances reusability. The stability of Cu^+^@COP underscores its potential as a practical alternative to existing CuAAC catalysts, expanding its applicability in green and industrial chemistry.

## 2 Results and Discussion

### 2.1 Synthesis and Structural Characterization of the Covalent Organic Polymer and Copper-Functionalized Systems

The COP was synthesized through a direct amidation reaction between pyridine-2,6-dicarboxylic acid and tetrakis­(4-aminophenyl)­methane^29^ under solvothermal conditions (Figure 1a). The resulting material was obtained as a light brown, insoluble solid in common solvents such as water, methanol, ethanol, *N,N*-dimethylformamide, dimethyl sulfoxide, *o*-xylene, tetrahydrofuran, toluene, chloroform, dichloromethane and *n*-hexane. The incorporation of copper species was carried out by treating COP with Cu­(II) acetate (50 mM) in aqueous solution, yielding Cu^**2**+^@COP, which was subsequently reduced to Cu^+^@COP using a solution of ascorbic acid (50 mM). The color evolution from light brown (COP) to green (Cu^**2**+^@COP) and finally to yellow-green (Cu^+^@COP) suggested a successful sequential coordination and reduction process (Figure 1b).

**Figure 1:**
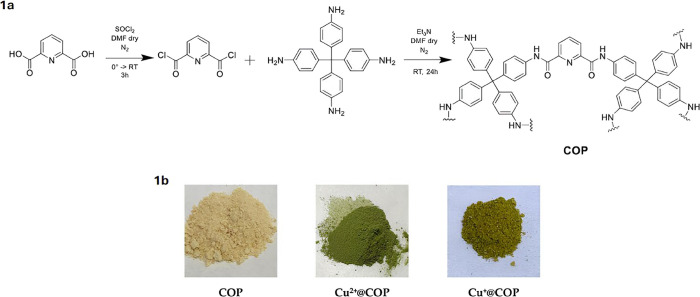
Synthesis and color evolution of the covalent organic polymer and its copper-functionalized derivatives. (a) Reaction scheme for the formation of COP via amidation between pyridine-2,6-dicarboxylic acid and tetrakis­(4-aminophenyl)­methane. (b) Color changes from COP (light brown) to Cu^2+^@COP (green) and Cu^+^@COP (yellow-green), indicative of successful copper coordination and reduction.

The powder X-ray diffraction (pXRD) analysis confirmed the amorphous nature of the polymer, as broad and diffuse diffraction patterns were observed rather than sharp peaks associated with crystalline order (Figure 2a). Thermal stability was evaluated using thermogravimetric analysis (TGA), which showed an initial weight loss below 150 °C attributed to adsorbed water and residual solvents; a minor decomposition stage at 250–350 °C consistent with partial degradation of pendant groups; and a major decomposition above 400 °C corresponds to backbone degradation. Copper coordination increases the onset temperature of thermal decomposition, consistent with literature on metal-cross-linked polymers^30^ (Figure 2b). The stability was only slightly affected by metal incorporation, as Cu^2+^@COP and Cu^+^@COP displayed similar decomposition profiles.

**Figure 2:**
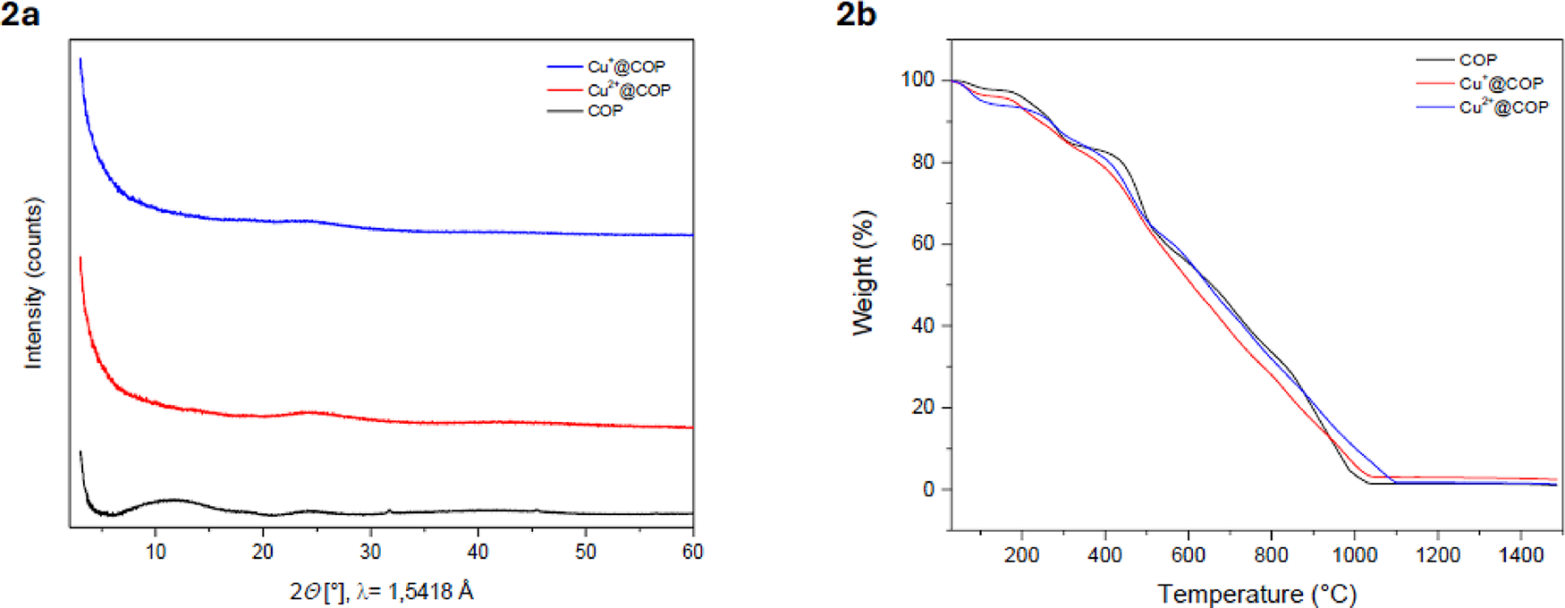
Physicochemical characterization of COP and copper-functionalized COPs. (a) pXRD patterns indicating the amorphous structure of all materials. (b) TGA thermograms showing thermal stability up to 500 °C with minor variations due to metal incorporation.

XPS measurements were carried out to determine the oxidation state of copper in Cu^+^@COP and Cu^2+^@COP. The Cu 2p_3__/__2_ spectra for both samples (Figure 3a) exhibited a primary component at 934.45 eV, accompanied by a 2-fold satellite in the range 940–945 eV, features characteristic of Cu­(II) species. The presence of these shakeup satellites, associated with *d* ^9^ unscreened core-ionized states, confirms the presence of Cu­(II) in both materials, in agreement with previous studies on copper oxides.^31^ In Cu^+^@COP, an additional component at 932.32 eV was observed, which lacks the characteristic Cu­(II) satellites and is consistent with Cu­(I) species.^32^ These results indicate that Cu­(I) and Cu­(II) coexist within the Cu^+^@COP framework, with partial oxidation of Cu­(I) likely occurring upon exposure to ambient conditions, as commonly reported for copper-based materials.^31^ Fourier-transform infrared (FT-IR) spectroscopy further confirmed metal coordination effects (Figure 3b). The pristine COP displayed characteristic amide vibrations, including a weak N–H stretching band at ∼3320 cm^–1^, the amide I (C=O stretching) band at ∼1665 cm^–1^, and the amide II (N–H bending) band at ∼1584 cm^–1^. Additional skeletal vibrations in the 1550–1300 cm^–1^ range were attributed to C–C and C–N stretching within the aromatic rings, with out-of-plane C–H bending at 812 and 748 cm^–1.^ ^33^ Upon copper coordination, the amide I band shifted from 1665 cm^–1^ to 1632 cm^–1^, indicating metal coordination at the carbonyl oxygen.^34–37^ The spectral region 1430–1300 cm^–1^ also exhibited changes, suggesting coordination involving nitrogen atoms from both pyridine and aniline-derived moieties. To further investigate the copper coordination environment, Raman spectroscopy was performed (Figure 3c). The free ligand exhibited intense fluorescence, preventing spectral acquisition, whereas Cu-based COP materials showed well-defined Raman bands. The spectra of Cu^+^@COP and Cu^2+^@COP were similar, with variations in the relative intensities of skeletal and C–N vibrational modes, suggesting differences in Cu­(I) and Cu­(II) coordination. The pyridine ring breathing mode was observed at ∼1004 cm^–1^ (Cu­(I)) and ∼1007 cm^–1^ (Cu­(II)), in agreement with literature reports.^38^ In the low-energy region, a distinct peak at ∼416 cm^–1^ was assigned to δ­(Cu–O–Cu) bending vibrations,^39^ whereas diagnostic Cu–N vibrations were not clearly detectable, possibly due to low intensity. Diffuse reflectance UV–vis spectroscopy provided additional insight into the electronic structure of the copper species (Figure 3d). The pristine polymer exhibited a strong absorption at 278 nm, attributed to π–π* transitions within the aromatic domains, a feature retained in both Cu^+^@COP and Cu^2+^@COP. In Cu^2+^@COP, an additional band at ∼660 nm was observed, corresponding to *d*–*d* transitions of Cu­(II), whereas in Cu^+^@COP, only a minor absorption was detected in this region, suggesting that a fraction of Cu­(II) persists due to oxidative processes.^40^

**Figure 3:**
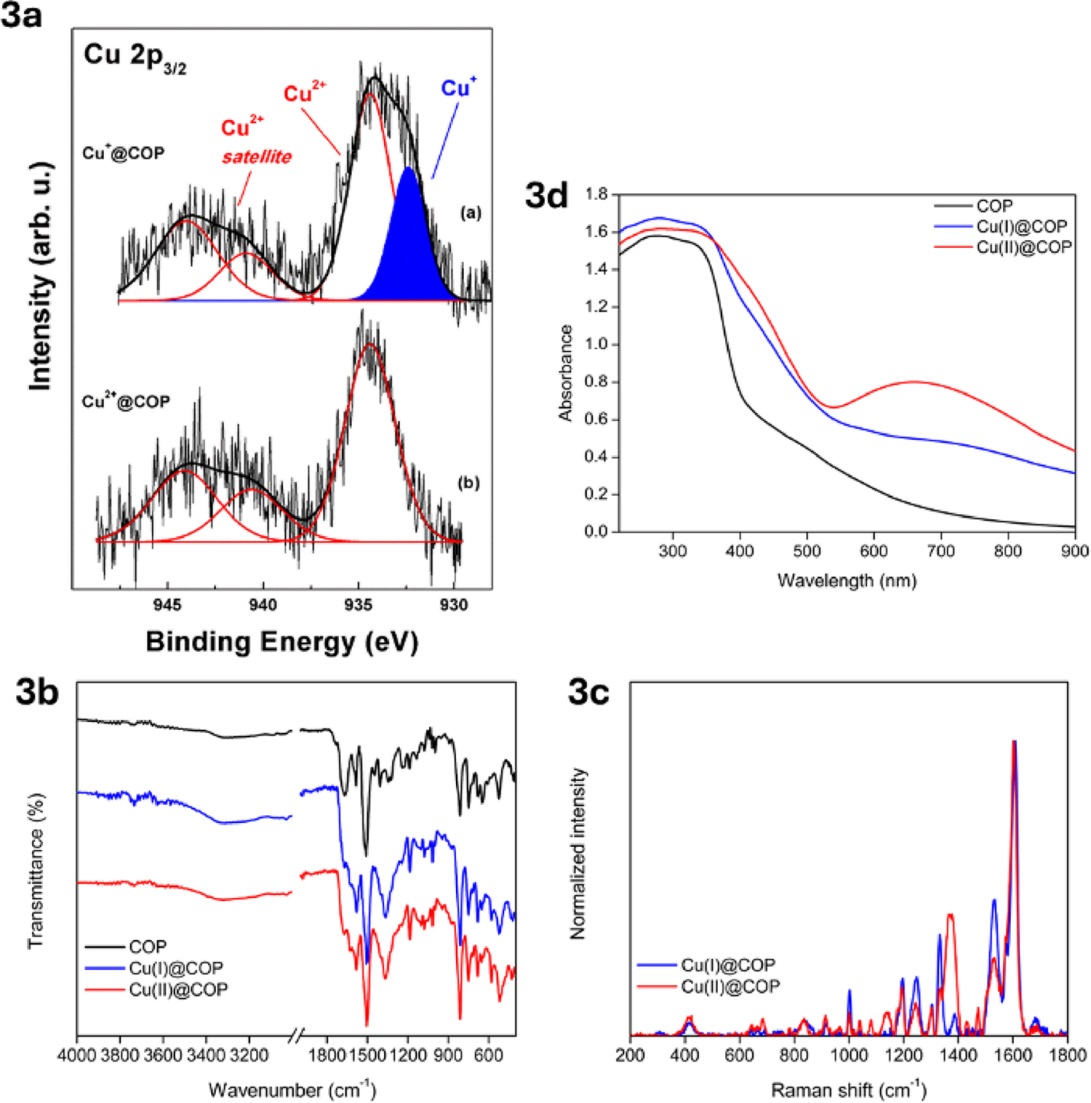
Spectroscopic analysis of Cu coordination in COP. (a) XPS Cu 2p_3__/__2_ spectra showing the presence of Cu­(I) and Cu­(II) species. (b) FT-IR spectra highlighting shifts in amide I band upon Cu coordination. (c) Raman spectra comparing vibrational features of Cu^+^@COP and Cu^2+^@COP. (d) Diffuse reflectance UV–vis spectra showing *d*–*d* transitions in Cu^2+^@COP and the preserved π–π* transitions in all samples.

ICP-OES analysis revealed a copper loading of 3.0 wt % in Cu^2+^@COP, which is maintained in Cu^+^@COP. Scanning electron microscopy (SEM) and atomic force microscopy (AFM) provided additional structural insights. The pristine polymer displayed a homogeneous and prevailingly flattened morphology (Figure 4a,d) with aggregated domains,^41^ while Cu^2+^@COP (Figure 4b,e) exhibited a distinct increase in surface roughness due to the formation of a multilevel organization process into spherical metal-rich nanostructures.

**Figure 4:**
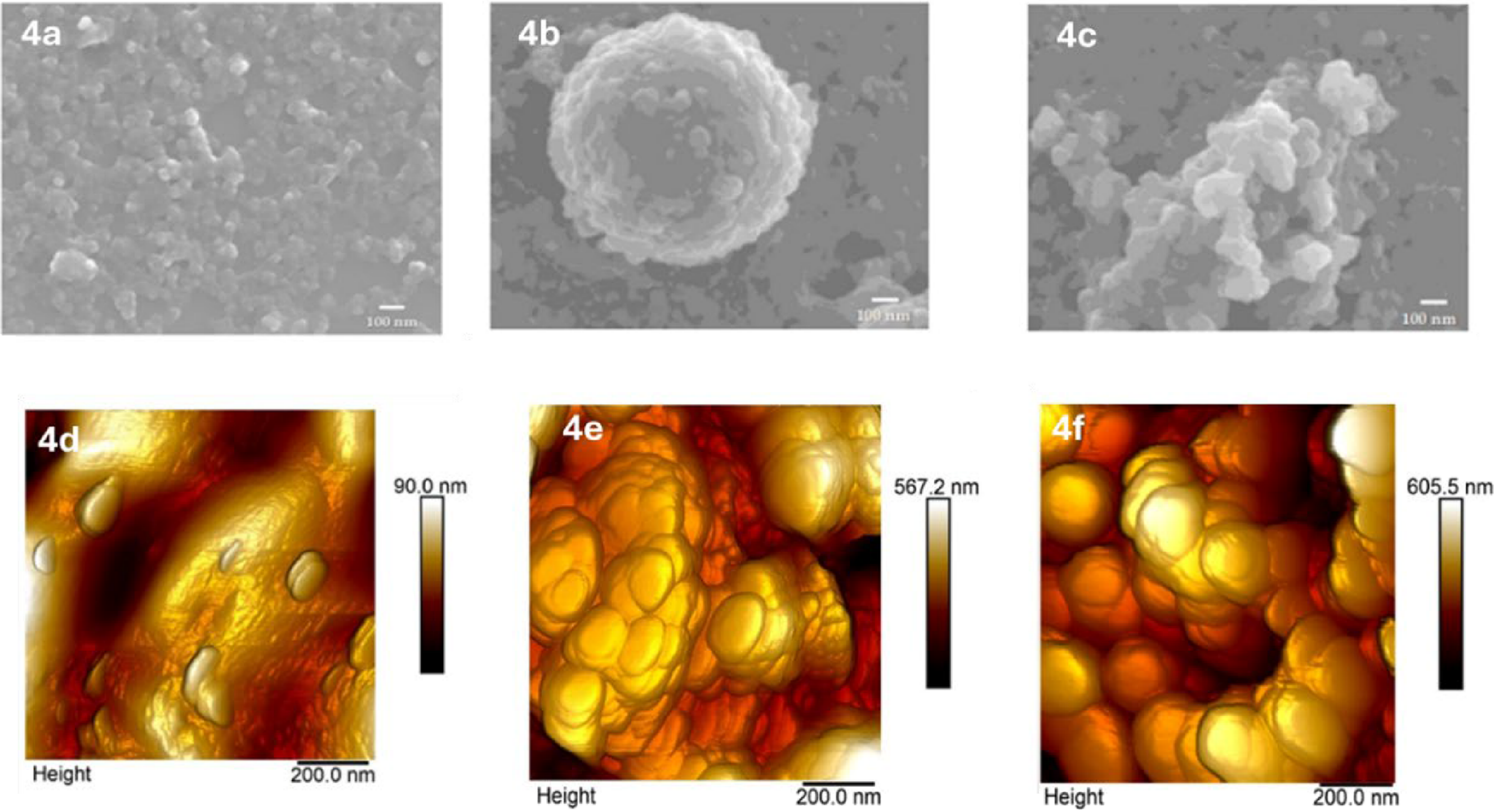
Morphological characterization of the catalysts. (a–c) SEM images of pristine COP, Cu^2+^@COP, and Cu^+^@COP. (d–f) Corresponding AFM images showing increased surface roughness upon copper incorporation and maintenance of morphology after reduction.

The reduction to Cu^+^@COP preserved these characteristic hierarchical assemblies (Figure 4c,f), suggesting that the overall integrity of the polymer was maintained throughout the metal coordination and reduction processes.

The exceptional stabilization of Cu­(I) within the COP framework and the complete suppression of leaching can be rationalized by considering the coordination environment provided by the polymer. In our system, the pyridine and amide functionalities act as multidentate N,O-donor ligands, forming chelating coordination to the copper centers. Similar stabilization effects have been observed in NHC-based polynuclear copper systems, where strong σ-donation from nitrogen ligands reduces the susceptibility of Cu­(I) to oxidation and disproportionation, even in aqueous environments.^42^ Likewise, pyridine-amide architectures have been reported to anchor Cu­(I) in a way that limits ligand exchange and restricts solvent access, as demonstrated in functionalized MOFs and COFs used for CuAAC catalysis.^4,8^ The cooperative effect of N,O-chelation not only modulates the electron density at the copper site but also sterically shields it from oxidative species, in analogy to enzyme-mimetic single-chain nanoparticles where intramolecular folding creates a protective pocket around the Cu­(I) center.^21,22^ In our COP, the amide carbonyl oxygen and the pyridine nitrogen likely form a bidentate coordination motif, which—combined with the rigid, cross-linked polymer network—prevents copper migration into the solution phase and thereby eliminates leaching. This immobilization mode is further supported by previous reports showing that covalent immobilization of Cu­(I) on multidentate nitrogen frameworks leads to both enhanced recyclability and retention of oxidation state under aerobic aqueous CuAAC conditions.^4,18^

### 2.2 Catalytic Performance in CuAAC Reactions

The catalytic activity of Cu^+^@COP was evaluated in the CuAAC between benzyl azide and phenylacetylene under mild conditions. To assess the role of copper oxidation state and the polymeric support, the reaction was performed using three different catalytic systems: (i) Cu^+^@COP, which does not require any external reducing agent; (ii) homogeneous CuSO_4_ in the presence of ascorbic acid, where continuous reduction of Cu­(II) to Cu­(I) is necessary to sustain catalytic activity; and (iii) Cu^2+^@COP with ascorbic acid, ensuring in situ reduction of Cu­(II) to Cu­(I) within the polymeric framework. This experimental setup allowed us to directly compare the efficiency of prereduced Cu­(I) species in Cu^+^@COP, which remain stabilized within the polymer matrix, against systems that require the continuous presence of a reducing agent to maintain Cu­(I) in its active state. The reaction catalyzed by homogeneous CuSO_4_ with ascorbic acid proceeded with low efficiency, yielding only 10% at room temperature after 24 h, as already described in a previously study.^43^ In contrast, Cu^2+^@COP in the presence of ascorbic acid exhibited improved activity, achieving around 40% both at room temperature and at 60 °C, suggesting that copper immobilization enhances catalytic performance but still requires a reducing agent to maintain the reduced Cu­(I) state. The most significant improvement was observed with Cu^+^@COP, which, despite the absence of ascorbic acid, provided a 95% yield under identical conditions, demonstrating that the prereduced Cu­(I) species remain catalytically active within the polymeric matrix without the need for continuous reduction.

It is noteworthy that the reaction proceeds with good yields under heterogeneous conditions in polar aprotic solvents such as ACN and THF. In these solvents, no reaction occurs in the homogeneous phase system (i.e., yield < 1%).

These results confirm that Cu^+^@COP operates efficiently in CuAAC reactions without requiring an external reducing agent, differentiating it from conventional homogeneous and heterogeneous Cu­(II)-based systems that necessitate chemical reduction to sustain Cu­(I) activity (Table 1).

**Table 1:**
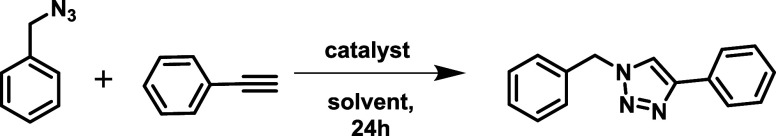
Catalytic Performance of Cu^+^@COP and Cu^2+^@COP in the CuAAC Reaction under Various Solvent Systems, Temperatures, and Catalyst Loadings (Based on Copper Content)

catalyst	loading (mol %)	solvent (v/v)	temperature (°C)	yield (%)
Cu^2+^@COP	1	ACN	R.T.	36
Cu^+^@COP	1	ACN	R.T.	23
homogeneous phase	1	ACN	R.T.	<1
Cu^2+^@COP	1	ACN	60	65
Cu^+^@COP	1	ACN	60	35
homogeneous phase	1	ACN	60	1
Cu^2+^@COP	1	toluene	R.T.	1
Cu^+^@COP	1	toluene	R.T.	<1
homogeneous phase	1	toluene	R.T.	<1
Cu^2+^@COP	1	THF	R.T.	19
Cu^+^@COP	1	THF	R.T.	17
homogeneous phase	1	THF	R.T.	<1
Cu^2+^@COP	1	H_2_O/^*t*^But (2:1)	R.T.	9
Cu^+^@COP	1	H_2_O/^*t*^But (2:1)	R.T.	35
homogeneous phase	1	H_2_O/^*t*^But (2:1)	R.T.	10
Cu^2+^@COP	1	H_2_O/^*t*^But (2:1)	60	40
Cu^+^@COP	1	H_2_O/^*t*^But (2:1)	60	60
homogeneous phase	1	H_2_O/^*t*^But (2:1)	60	39
**Cu** ^**2+**^ **@COP**	**5**	**H**_**2**_ **O/** *t-* **BuOH**(2:1)	**R.T.**	**46**
**Cu** ^**+**^ **@COP**	**5**	**H**_**2**_ **O/** *t-* **BuOH**(2:1)	**R.T.**	**95**
**homogeneous phase**	**5**	**H**_**2**_ **O/** *t-* **BuOH**(2:1)	**R.T.**	**55**
Cu^2+^@COP	1	MeOH	R.T.	45
Cu^+^@COP	1	MeOH	R.T.	42
Homogeneous Phase	1	MeOH	R.T.	39
Cu^2+^@COP	1	H_2_O/MeOH (1:1)	R.T.	34
Cu^+^@COP	1	H_2_O/MeOH (1:1)	R.T.	36
homogeneous phase	1	H_2_O/MeOH (1:1)	R.T.	39
Cu^2+^@COP	1	H_2_O/MeOH (1:1)	60	43
Cu^+^@COP	1	H_2_O/MeOH (1:1)	60	75
homogeneous phase	1	H_2_O/MeOH (1:1)	60	60
Cu^2+^@COP	1	neat	R.T.	<1
Cu^+^@COP	1	neat	R.T.	<1
homogeneous phase	1	neat	R.T.	<1

The effect of substrate structure on catalytic performance was further explored by evaluating a series of azides and alkynes with varying electronic and steric properties (Table 2).

**Table 2:**
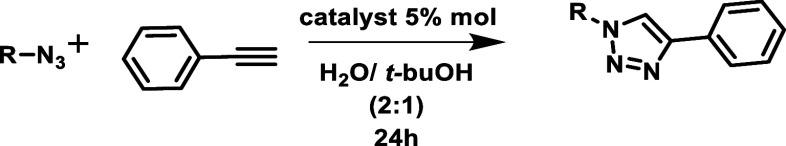

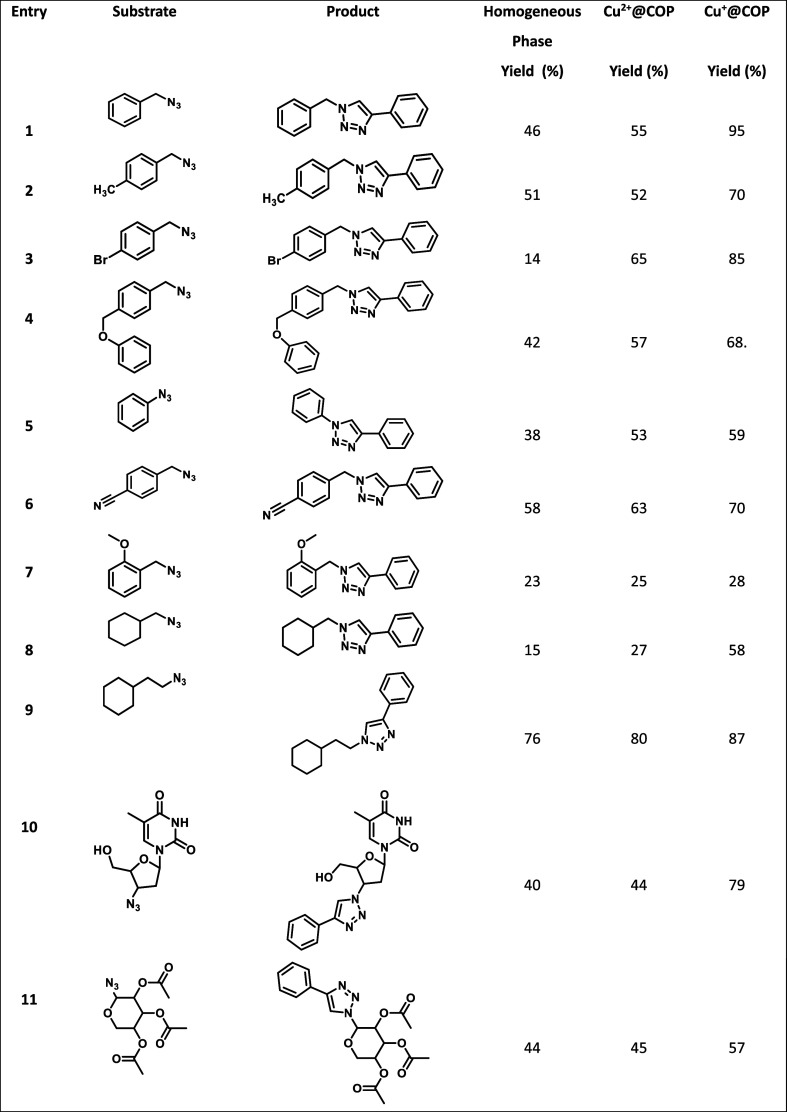
Substrate Scope in CuAAC Reactions Catalyzed by Cu^+^@COP, Cu^2+^@COP, and Homogeneous Systems

Electron-withdrawing substituents on the azide moiety led to an increase in reaction rates, likely due to the increased electrophilicity of the azide group. In contrast, sterically hindered azides exhibited a decrease in reactivity, particularly in ortho-substituted derivatives, which likely suffer from reduced accessibility to the catalytic sites. Aliphatic azides were found to react more efficiently than their aromatic counterparts, indicating that steric and solubility factors play a significant role in governing reaction kinetics. To verify the heterogeneous nature of the catalyst, a leaching test was conducted by removing Cu^+^@COP from the reaction medium after an initial reaction period. The reaction ceased upon catalyst removal, confirming that catalysis was occurring exclusively at the solid-phase active sites and not due to dissolved copper species. When compared to reported heterogeneous CuAAC catalysts, Cu^+^@COP shows competitive activity under mild, aqueous conditions without the need for added reducing agents. For instance, Cu-incorporated COFs often require organic cosolvents to achieve high yields, and leaching is commonly observed, as in the case of amide-linked COFs for CuAAC.^44^ Similarly, MOF-based systems, such as mechanochemically activated Cu-MOFs, reach yields above 90% only under organic solvent conditions and prolonged reaction times.^18^ In contrast, Cu^+^@COP achieves up to 95% yield in H_2_O/*t*-BuOH within 24 h, maintaining full copper content after reaction as confirmed by ICP-OES, and preserving its oxidation state (namely, maintaining catalytic activity through several cycles). This performance, together with complete suppression of leaching, highlights the combined effect of the rigid, cross-linked polymer framework and the multidentate N,O-coordination environment in sustaining Cu­(I) catalysis under operationally simple, green conditions.

### 2.3 Recyclability and Stability of Cu^+^@COP

A crucial advantage of Cu^+^@COP is its high recyclability and operational stability. The catalyst was recovered via simple filtration, washed, and reused over multiple cycles without additional treatment. After four consecutive reaction runs (i.e., model reaction presented in the scheme in Table 1), the yields remained consistently above 90%, decreasing at 85% from the fourth cycle (Figure 5). To further investigate the structural integrity of the catalyst upon reuse, pXRD analysis of Cu^+^@COP was carried out after 1, 2, 3, and 4 catalytic cycles. The diffraction patterns remained unchanged compared to the fresh material, confirming that the polymeric framework is preserved during the catalytic process and does not undergo detectable structural degradation (see Supporting Information). Moreover, the consistent high yields observed over multiple catalytic cycles indicate that the Cu­(I) species remain stable and active within the polymeric matrix throughout the reaction process, without undergoing significant oxidation or leaching.

**Figure 5:**
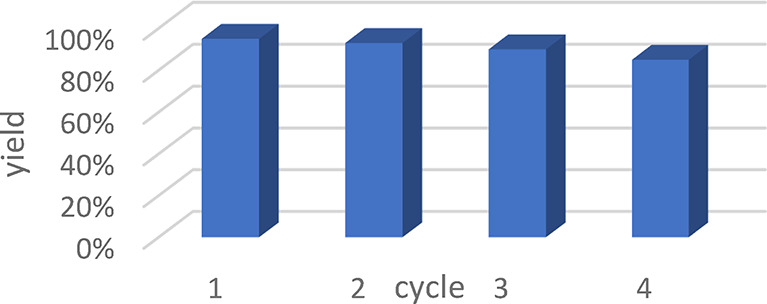
Catalyst recyclability performance of Cu^+^@COP. Recycling test for three consecutive catalytic cycles shows consistent yields above 90%, with a decrease at 80% from the fourth cycle.

To assess whether any significant copper loss occurred during the catalytic process, ICP-OES analysis was performed directly on the recovered solid catalyst after multiple reaction cycles. The copper content remained unchanged within experimental error, with 3 wt % measured before and after reuse, indicating that no detectable leaching of copper occurred during the reaction. The constancy of metal suggests a strong retention of copper species within the polymeric network, further confirming the stability of the catalyst.

## 3 Conclusions

This study presents Cu^+^@COP as an efficient and recyclable heterogeneous catalyst for CuAAC reactions, operating without the need for external reducing agents. Unlike conventional systems that require continuous Cu­(II) reduction, Cu^+^@COP maintains Cu­(I) in its active form within the polymeric matrix, enabling high catalytic efficiency and simplified reaction conditions.

A key aspect of this work is that the oxidation state of copper in a heterogeneous CuAAC system has been directly analyzed. XPS confirmed the coexistence of Cu­(I) and Cu­(II) in Cu^+^@COP, while ICP-MS showed no significant metal leaching, ensuring long-term catalyst stability. Compared to CuSO_4_ and Cu^2+^@COP, which required ascorbic acid for sustained activity, Cu^+^@COP achieved superior performance (95% yield) under identical conditions without any reductant.

The combination of high efficiency, stability, and elimination of metal contamination risks makes Cu^+^@COP a practical alternative for CuAAC catalysis, with potential applications in organic synthesis and materials science. The results highlight the importance of monitoring oxidation states in heterogeneous copper catalysts and provide a basis for future developments in CuAAC systems.

## 4 Experimental Section

### 4.1 Chemicals and Reagents

All solvents and reagents used for the synthesis of COP, Cu^2+^@COP, Cu^+^@COP, and in the CuAAC “click” reaction were purchased from Merck Life Science (Darmstadt, Germany) and used without further purification unless otherwise specified. Precoated silica gel F254 sheets from Merck Life Science were used for reaction monitoring by thin-layer chromatography (TLC).

### 4.2 Instrumentation

#### 4.2.1 Spectroscopic and Structural Characterization

##### 4.2.1.1 Nuclear Magnetic Resonance (NMR)

^1^H and ^13^C NMR spectra were recorded using a Bruker spectrometer in deuterated chloroform (CDCl_3_), deuterated methanol (MeOD), or deuterated dimethyl sulfoxide (DMSO). ^1^H NMR spectra were acquired at 400 MHz, and ^13^C NMR at 100 MHz.

##### 4.2.1.2 Fourier Transform Infrared Spectroscopy (FT-IR)

FT-IR spectra were recorded using a Jasco FT/IR-6800 spectrometer equipped with an ATR (Attenuated Total Reflectance) accessory. Spectra were collected in the range 4000–650 cm^–1^.

##### 4.2.1.3 Raman Spectroscopy

Raman spectra were obtained at room temperature using an inVia Renishaw micro-Raman spectrometer equipped with an air-cooled CCD detector and super-Notch filters. A 514 nm Ar^+^ laser was used with a 20× objective.

#### 4.2.2 Microscopy and Surface Analysis

##### 4.2.2.1 Scanning Electron Microscopy (SEM)

SEM images were obtained using a JEOL JSM-7100 microscope operating at 5 kV. Samples were drop-casted onto a silicon substrate for analysis.

##### 4.2.2.2 Atomic Force Microscopy (AFM)

AFM analysis was performed using a Bruker MultiMode 8 AFM microscope equipped with a Nanoscope V controller in Tapping Mode in air. Commercial RTESPA-150 silicon cantilevers (characterized by resonance frequency 150 kHz and nominal spring constant 6 N/m) with a tip radius of 8 nm were used. The scan size area was 1 × 1 μm. Images of 512 × 512 pixels were collected and elaborated using the Nanoscope Analysis 1.8 software.

#### 4.2.3 Thermal and Spectroscopic Analysis

##### 4.2.3.1 Powder X-ray Diffraction (pXRD)

pXRD patterns were recorded using a MiniFlex 600 diffractometer equipped with Cu Kα radiation. Data were collected in the 3–60° 2θ range, with a 0.02° step width.

##### 4.2.3.2 Thermogravimetric Analysis (TGA)

TGA was conducted using a TA Instruments SDT 650 analyzer in a dry nitrogen atmosphere (50 mL/min flow rate). Samples were heated from 30 to 1500 °C at 20 °C/min using alumina crucibles.

##### 4.2.3.3 UV–Vis Spectroscopy

Diffuse reflectance UV–vis spectra were recorded using a Shimadzu UV-2600i spectrometer equipped with an integrating sphere (ISR-2600Plus). Measurements were performed in the range 220–900 nm with a data interval of 0.1 nm.

#### 4.2.4 Elemental and Oxidation State Analysis

##### 4.2.4.1 Inductively Coupled Plasma Optical Emission Spectroscopy (ICP-OES)

Copper content was determined using an iCAP 7200 ICP-OES Duo from Thermo Fisher Scientific, operated in dual view (radial and axial configuration) (see Supporting Information).

##### 4.2.4.2 X-ray Photoelectron Spectroscopy (XPS)

XPS measurements were performed using an Omicron NanoTechnology Multiprobe MXPS system with a Mg Kα (hν = 1253.6 eV) X-ray source, operating the anode at 14 kV and 16 mA. The analyzer pass energy was set to 20 eV, and a takeoff angle of 21° with respect to the sample surface normal was adopted.

##### 4.2.4.3 Synthesis of Covalent Organic Polymer (COP)

COP was synthesized through a solvothermal procedure starting from pyridine-2,6-dicarboxylic acid and tetrakis­(4-aminophenyl)­methane. In a 250 mL round-bottom flask, pyridine-2,6-dicarboxylic acid (2.10 mmol) was dissolved in *N,N*-dimethylformamide (DMF, 4 mL), and thionyl chloride (2.10 mmol) was added dropwise at 0 °C under stirring. The mixture was allowed to react at room temperature for 3 h. Separately, tetrakis­(4-aminophenyl)­methane (1.05 mmol) and triethylamine (3.15 mmol) were dissolved in DMF (2 mL) and added to the activated acid solution. The reaction was stirred at room temperature for 24 h under a nitrogen atmosphere. The resulting polymer was precipitated by adding a saturated NaCl solution, filtered, washed with deionized water, and dried at 100 °C under vacuum.

##### 4.2.4.4 Synthesis of Cu^2+^@COP

The polymer (100 mg) was suspended in a 50 mM aqueous solution of Cu­(OAc)_2_ (20 mL) and stirred at room temperature for 24 h. The solid was collected by filtration, washed with deionized water and ethanol, and dried at 100 °C under vacuum to obtain Cu^2+^@COP.

##### 4.2.4.5 Synthesis of Cu^+^@COP

Cu^2+^@COP (100 mg) was added to a 50 mM aqueous solution of ascorbic acid (20 mL) under a nitrogen atmosphere. The suspension was stirred at room temperature for 3 h, filtered, washed with deionized water, and dried at 100 °C under inert atmosphere to yield Cu^+^@COP.

##### 4.2.4.6 General Procedure for CuAAC Reactions

A 25 mL round-bottom flask equipped with a magnetic stirring bar was charged with azide (0.5 mmol), dissolved in a mixture of H_2_O/*t*-BuOH (2:1, 2 mL). Copper sulfate (0.02 mmol), ascorbic acid (0.09 mmol), and phenylacetylene (0.5 mmol; ρ = 0.930 g/cm^3^) were then added sequentially. The reaction mixture was stirred at room temperature under nitrogen atmosphere until completion. For reactions using Cu^+^@COP or Cu^2+^@COP, the corresponding catalyst was added (5 mol % based on copper content). In the case of Cu^2+^@COP, ascorbic acid (0.09 mmol) was additionally introduced to the mixture. After stirring at room temperature, the reaction was quenched by filtration to recover the heterogeneous catalyst. The filtrate was extracted with EtOAc (3 × 20 mL), and the combined organic layers were dried over anhydrous Na_2_SO_4_, filtered, and concentrated under reduced pressure to afford the crude product. The residue was purified by recrystallization from *n*-hexane to yield the desired product as a white powder. Yields (%) are reported in Table 2.

#### 4.2.5 Cautionary Note

Organic azides can be explosive and toxic; they should always be handled in dilute solution, on small scale, behind a blast shield, and with appropriate PPE. Avoid contact with strong acids and dispose of azide-containing waste according to institutional safety protocols.

#### 4.2.6 Characterization

##### 4.2.6.1 1-Benzyl-4-phenyl-1H-1,2,3-triazole (Entry 1)^45^

White crystalline solid, 112 mg (95%).

**1H NMR (MeOD**_**4**_, **400 MHz):** δ 7.82 (d, *J* = 7.4 Hz, 2H), 7.69 (s, 1H), 7.52–7.31 (m, 7H), 5.60 (s, 2H).

**13C­{H}- NMR (101 MHz, MeOD**_**4**_ **):** δ 148.2, 134.7, 130.5, 129.1, 129.0–128.7, 128.1, 128.0, 127.1, 125.7, 119.5, 54.2.

##### 4.2.6.2 1-(4-Methylbenzyl)-4-phenyl-1H-1,2,3-triazole (Entry 2)^46^

White crystalline solid, 87 mg (70%)

**1H NMR (400 MHz, Chloroform-*d*):** δ 7.81 (d, *J* = 7.5 Hz, 2H), 7.66 (s, 1H), 7.48–7.19 (m, 9H), 5.56 (s, 2H), 2.38 (s, 3H).

**13C­{H}- NMR (101 MHz, Chloroform-*d*):** δ 148.1, 138.7, 131.6, 130.5, 129.8, 128.8, 128.1, 125.7, 54.1, 21.1.

##### 4.2.6.3 1-(4-Bromobenzyl)-4-phenyl-1H-1,2,3-triazole (Entry 3)^46^

White crystalline solid, 134 mg (85%).

**1H NMR (400 MHz, Chloroform-*d*):** δ 7.80 (d, *J* = 7.6 Hz, 2H), 7.66 (s, 1H), 7.52 (d, *J* = 8.3 Hz, 2H), 7.41 (t, *J* = 7.6 Hz, 2H), 7.33 (t, *J* = 7.3 Hz, 1H), 7.19 (d, *J* = 8.3 Hz, 2H), 5.54 (s, 2H).

**13C­{H}- NMR (101 MHz, Chloroform-*d*):** δ 133.7, 132.3, 130.3, 129.6, 128.8, 128.3, 125.73, 122.9, 53.7.

##### 4.2.6.4 1-(4-(Phenoxymethyl)­benzyl)-4-phenyl-1H-1,2,3-triazole (Entry 4)^47^

White crystalline solid,116 mg (68%).

**1H NMR (400 MHz, Chloroform-*d*):** δ 7.82 (d, *J* = 8.0 Hz, 2H), 7.66 (s, 1H), 7.50–7.22 (m, 10H), 7.01 (d, *J* = 8.3 Hz, 2H), 5.54 (s, 2H), 5.10 (s, 2H).

**13C­{H}- NMR (101 MHz, Chloroform-*d*):** δ 159.1, 148.1, 136.6, 130.6, 129.7, 128.8, 128.6, 128.1,127.4, 126.9, 125.7, 119.3, 115.4, 70.1, 53.7.

##### 4.2.6.5 1,4-Diphenyl-1H-1,2,3-triazole (Entry 5)^48^

White crystalline solid, 65 mg (59%).

**1H NMR (400 MHz, Chloroform-*d*):** δ 8.23 (s, 1H), 7.99–7.90 (m, 2H), 7.89–7.78 (m, 2H), 7.58 (t, *J* = 7.8 Hz, 2H), 7.53–7.44 (m, 3H), 7.40 (t, *J* = 7.4 Hz, 1H).

**13C­{H}- NMR (101 MHz, Chloroform-*d*):** δ 148.4, 137.0, 130.2, 129.8, 128.9, 128.8, 128.4, 125.8, 120.5, 117.6.

##### 4.2.6.6 4-((4-Phenyl-1H-1,2,3-triazol-1-yl)­methyl)­benzonitrile (Entry 6)^46^

White crystalline solid, 91 mg (70%).

**1H NMR (400 MHz, Chloroform-*d*):** δ 7.84 (d, *J* = 8.1 Hz, 2H), 7.75 (s, 1H), 7.71 (d, *J* = 8.1 Hz, 2H), 7.52–7.32 (m, 5H), 5.68 (s, 2H).

**13C­{H}- NMR (101 MHz, Chloroform-*d*):** δ 148.6, 139.9, 132.9, 130.1, 128.9, 128.5, 128.37, 125.7, 119.7, 118.1, 112.8, 53.4.

##### 4.2.6.7 1-(2-Methoxybenzyl)-4-phenyl-1H-1,2,3-triazole (Entry 7)

White crystalline solid, 37 mg (28%).

**1H NMR (400 MHz, Chloroform-*d*):** δ 7.83 (d, *J* = 7.5 Hz, 2H), 7.74 (s, 1H), 7.51–7.19 (m, 6H), 7.06–6.87 (m, 2H), 5.62 (s, 2H), 3.91 (s, 3H).

**13C­{H}- NMR (101 MHz, Chloroform-** ***d*** **):** δ. 157.1, 130.6, 130.3, 128.7, 128.0, 125.7, 122.9, 121.0, 110.8, 55.5, 49.2.

HRMS (ESI) *m*/*z*: [M + H]^+^ calcd. for C_16_H_16_N_3_O 266.1288; found: 266.1290.

##### 4.2.6.8 1-(Cyclohexylmethyl)-4-phenyl-1H-1,2,3-triazole (Entry 8)

White amorphous solid, 70 mg (58%).

**1H NMR (400 MHz, DMSO-*d*_6_):** δ 8.56 (s, 1H), 7.93–7.76 (m, 2H), 7.45 (t, *J* = 7.6 Hz, 2H), 7.33 (t, *J* = 7.4 Hz, 1H), 4.25 (d, *J* = 7.1 Hz, 2H), 1.87 (m, 1H), 1.75–1.43 (m, 4H), 1.31–1.14 (m, 4H), 1.00 (m, 2H).

**13C­{H}- NMR (101 MHz, Chloroform-*d*):** δ 147.5, 130.7, 128.8, 128.0, 119.9, 56.6, 38.8, 30.5, 26.0, 25.5.

HRMS (ESI) *m*/*z*: [M + H]^+^ calcd. for C_15_H_20_N_3_ 242.1652; found: 242.1653.

##### 4.2.6.9 1-(2-Cyclohexylethyl)-4-phenyl-1H-1,2,3-triazole (Entry 9)

White amorphous solid, 111 mg (87%).

**1H NMR (400 MHz, DMSO-*d*_6_):** δ 8.46 (s, 1H), 7.72 (d, *J* = 7.1 Hz, 2H), 7.31 (t, *J* = 7.6 Hz, 2H), 7.19 (t, *J* = 7.4 Hz, 1H), 4.28 (t, *J* = 7.4 Hz, 2H −CH2−), 1.73–1.36 (m, 7H), 1.12–0.66 (m, 6H).

**13C­{H}- NMR (101 MHz, Chloroform-*d*):** δ 147.7, 130.7, 128.8, 128.0, 125.6, 119.3, 48.2, 37.7, 34.9, 32.9, 26.3, 26.0.

HRMS (ESI) *m*/*z*: [M + H]^+^ calcd. for C_16_H_22_N_3_ 256.1808; found: 256.1809.

##### 4.2.6.10 1-(5-(Hydroxymethyl)-4-(4-phenyl-1H-1,2,3-triazol-1-yl)-2,5-dihydrofuran-2-yl)-5-methylpyrimidine-2,4­(1H,3H)-dione (Entry 10)

White amorphous solid, 146 mg (79%).

**1H NMR (400 MHz, DMSO-*d*_6_):** δ 11.38 (s, 1H), 8.79 (s, 1H), 7.94 (d, *J* = 8.2 Hz, 2H), 7.85 (s, 1H), 7.47 (t, *J* = 7.6 Hz, 2H), 7.41–7.24 (m, 1H), 6.46 (t, *J* = 6.6 Hz, 1H), 5.42 (dt, *J* = 8.7, 5.4 Hz, 1H), 5.31 (t, *J* = 5.2 Hz, 1H), 4.30 (dt, *J* = 5.5, 3.6 Hz, 1H), 3.80–3.55 (m, 2H), 2.88–2.62 (m, 2H), 1.83 (s, 3H).

**13C­{H}- NMR (101 MHz, DMSO-*d*_6_):** δ 164.2, 150.9, 147.0, 136.7, 131.0, 129.4, 128.4, 125.6, 121.4, 110.1, 84.9, 84.3, 61.2, 59.8, 37.6, 15.6, 12.7.

HRMS (ESI) *m*/*z*: [M + Na]^+^ calcd. for C_18_H_19_N_5_O_4_Na 392.1329; found: 392.1329.

##### 4.2.6.11 2-(4-Phenyl-1H-1,2,3-triazol-1-yl)­tetrahydro-2H-pyran-3,4,5-triyl Triacetate (Entry 11)

White amorphous solid, 115 mg (57%).

**1H NMR (400 MHz, Chloroform-*d*):** δ 7.90 (s, 1H), 7.76 (d, *J* = 7.3 Hz, 2H), 7.37 (t, *J* = 7.5 Hz, 2H), 7.29 (t, *J* = 7.3 Hz, 1H), 5.80–5.75 (m, 1H), 5.43–5.34 (m, 2H), 5.12 (dq, *J* = 9.7, 5.5 Hz, 1H), 4.26 (dd, *J* = 11.6, 5.7 Hz, 1H), 3.60–3.52 (m, 1H), 2.02 (s, 3H), 2.00 (s, 3H). 1.83 (s, 3H).

**13C­{H}- NMR (101 MHz, Chloroform-*d*):** δ 169.9, 169.8, 169.1, 148.4, 130.2, 129.9, 129.1, 128.9, 128.5, 125.9, 117.6, 86.4, 72.1, 70.3, 68.4, 65.6, 20.6, 20.6, 20.2.

HRMS (ESI) *m*/*z*: [M + Na]^+^ calcd. for C_19_H_21_N_3_O_7_ Na 426.1272; found: 426.1273.

### 4.3 Recycling and Reusability Tests

The recyclability of Cu^+^@COP was assessed over four consecutive cycles. After each reaction, the catalyst was recovered by filtration, washed with ethanol and deionized water, and dried under vacuum at 100 °C for 5 h before reuse.

## Supplementary Material



## Data Availability

The data underlying this study are available in the published article and its Supporting Information.

## Notes

General procedure, characterization data, NMR spectra, pXRD spectra safter each catalysis cycle, and inductively coupled plasma optical emission spectroscopy (ICP-OES) (PDF)
