# Supplementary material for: Copper(I)-Anchoring Covalent Organic Polymer for Heterogeneous CuAAC Reaction without Reducing Agents and Copper Leaching
Source: ACS Org Inorg Au. 2025 Sep 25;5(6):507–17. doi: 10.1021/acsorginorgau.5c00067 (PMC12679302; doi:10.1021/acsorginorgau.5c00067)
Supplement: Supplementary file 1 [file gg5c00067_si_001.pdf]

Supporting Information

Copper(I)- Anchoring Covalent Organic Polymer for Heterogeneous CuAAC Reaction Without Reducing Agents and Copper Leaching

Maria Aurora Guarducci, [a] Simone Manetto,[a] Andrea Giacomo Marrani,[b] Francesco Amato,[b] Paolo Guglielmi,[a] Michele Coluccia, [a] Antonella Fontana,[c,d] Serena Pilato,[c,d] Claudio Villani,[a] Alessia Ciogli,[a] and Giulia Mazzoccanti*[a]

[a] Dr. M.A. Guarducci, Dr. S. Manetto, Dr. P. Guglielmi, Dr. Michele Coluccia, Prof. C. Villani Prof. A. Ciogli, Dr.G. Mazzoccanti,
Department of Chemistry and Technologies of drugs
Sapienza University of Rome
pl.e A. Moro 5, 00185 Roma, Italy
Email: giulia.mazzoccanti@uniroma1.it

[b] Prof. A .G. Marrani, Dr. F. Amato
Department of Chemistry
Sapienza University of Rome
pl.e A. Moro 5, 00185 Roma, Italy

[c] Prof. A. Fontana, Dr. S. Pilato
UdA-Tech Lab,
"G. d'Annunzio" University of Chieti-Pescara
66100 Chieti, Italy

[d] Prof. A. Fontana, Dr. S. Pilato
Department of Pharmacy,
"G. d'Annunzio" University of Chieti-Pescara,
66100 Chieti, Italy

Supporting Information

General procedure, Characterization Data, NMR spectra, pXRD spectra safter each catalysis cycle, Inductively Coupled Plasma Optical Emission Spectroscopy (ICP-OES)

Table of contents

General procedure……………………………………………………S1
Characterization Data…………………………………………………S1
NMR spectra……………………………………………………………S7
pXRD spectra …………………………………………………………S29
Inductively Coupled Plasma Optical Emission Spectroscopy (ICP-OES)………………S30

General Procedure for CuAAC Reactions

A 25 mL round bottom flask equipped with a magnetic stirring bar was charged with azide (0.5 mmol), dissolved in a mixture of H_2O/t-BuOH (2:1, 2 mL). Copper sulfate (0.02 mmol), ascorbic acid (0.09 mmol), and phenylacetylene (0.5 mmol; d = 0.930 g/cm^3) were then added sequentially. The reaction mixture was stirred at room temperature under nitrogen atmosphere until completion. For reactions using Cu^+@COP or Cu^{2+}@COP, the corresponding catalyst was added (5 mol% based on copper content). In the case of Cu^{2+}@COP, ascorbic acid (0.09 mmol) was additionally introduced to the mixture. After stirring at room temperature, the reaction was quenched by filtration to recover the heterogeneous catalyst. The filtrate was extracted with EtOAc (3 × 20 mL), and the combined organic layers were dried over anhydrous Na_2SO_4, filtered, and concentrated under reduced pressure to afford the crude product. The residue was purified by recrystallization from n-hexane to yield the desired product as a white powder. Yields are reported in Table 2.

Cautionary Note

Organic azides can be explosive and toxic; they should always be handled in dilute solution, on small scale, behind a blast shield, and with appropriate PPE. Avoid contact with strong acids and dispose of azide-containing waste according to institutional safety protocols.

Characterization data

1-benzyl-4-phenyl-1H-1,2,3-triazole (entry 1)[45]

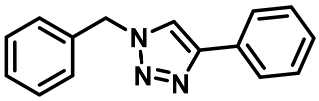

Chemical Formula: $C_{15}H_{13}N_3$ White crystalline solid, 112 mg (95%).

1H NMR (MeOD$_4$, 400 MHz): δ 7.82 (d, J = 7.4 Hz, 2H), 7.69 (s, 1H), 7.52 – 7.31 (m, 7H), 5.60 (s, 2H).

13C{H}- NMR (101 MHz, MeOD$_4$): δ 148.2, 134.7, 130.5, 129.1, 129.0 – 128.7, 128.1, 128.0, 127.1, 125.7, 119.5, 54.2.

1-(4-methylbenzyl)-4-phenyl-1H-1,2,3-triazole (entry 2)[46]

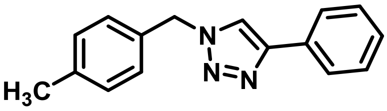

White crystalline solid, 87 mg (70%)

1H NMR (400 MHz, Chloroform-d): δ 7.81 (d, J = 7.5 Hz, 2H), 7.66 (s, 1H), 7.48 – 7.19 (m, 9H), 5.56 (s, 2H), 2.38 (s, 3H).

13C{H}- NMR (101 MHz, Chloroform-d): δ 148.1, 138.7, 131.6, 130.5, 129.8, 128.8, 128.1, 125.7, 54.1, 21.1.

1-(4-bromobenzyl)-4-phenyl-1H-1,2,3-triazole (entry 3)[46]

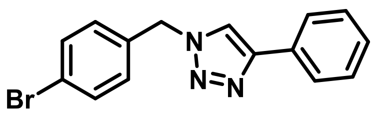

Chemical Formula: $C_{15}H_{12}BrN_3$ White crystalline solid, 134 mg (85%).

1H NMR (400 MHz, Chloroform-d): δ 7.80 (d, J = 7.6 Hz, 2H), 7.66 (s, 1H), 7.52 (d, J = 8.3 Hz, 2H), 7.41 (t, J = 7.6 Hz, 2H), 7.33 (t, J = 7.3 Hz, 1H), 7.19 (d, J = 8.3 Hz, 2H), 5.54 (s, 2H).

13C{H}- NMR (101 MHz, Chloroform-d): δ 133.7, 132.3, 130.3, 129.6, 128.8, 128.3, 125.73, 122.9, 53.7.

1-(4-(phenoxymethyl)benzyl)-4-phenyl-1H-1,2,3-triazole (entry 4)[47]

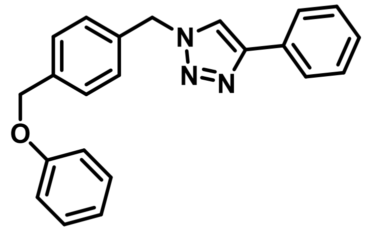

Chemical Formula: $C_{22}H_{19}N_3O$ White crystalline solid,116 mg (68%).

1H NMR (400 MHz, Chloroform-d): δ 7.82 (d, J = 8.0 Hz, 2H), 7.66 (s, 1H), 7.50 – 7.22 (m, 10H), 7.01 (d, J = 8.3 Hz, 2H), 5.54 (s, 2H), 5.10 (s, 2H).

13C{H}- NMR (101 MHz, Chloroform-d): δ 159.1, 148.1, 136.6, 130.6, 129.7, 128.8, 128.6, 128.1,127.4, 126.9, 125.7, 119.3, 115.4, 70.1, 53.7.

1,4-diphenyl-1H-1,2,3-triazole (entry 5)[48]

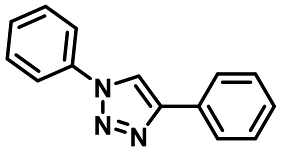

Chemical Formula: $C_{14}H_{11}N_3$ White crystalline solid, 65 mg (59%).

1H NMR (400 MHz, Chloroform-d): δ 8.23 (s, 1H), 7.99 – 7.90 (m, 2H), 7.89 – 7.78 (m, 2H), 7.58 (t, J = 7.8 Hz, 2H), 7.53 – 7.44 (m, 3H), 7.40 (t, J = 7.4 Hz, 1H).

13C{H}- NMR (101 MHz, Chloroform-d): δ 148.4, 137.0, 130.2, 129.8, 128.9, 128.8, 128.4, 125.8, 120.5, 117.6.

4-((4-phenyl-1H-1,2,3-triazol-1-yl)methyl)benzonitrile (entry 6)[46]

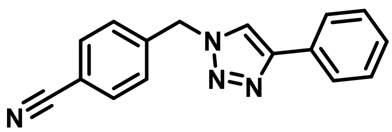

Chemical Formula: $C_{16}H_{12}N_4$ White crystalline solid, 91 mg (70%).

1H NMR (400 MHz, Chloroform-d): δ 7.84 (d, J = 8.1 Hz, 2H), 7.75 (s, 1H), 7.71 (d, J = 8.1 Hz, 2H), 7.52 – 7.32 (m, 5H), 5.68 (s, 2H).

13C{H}- NMR (101 MHz, Chloroform-d): δ 148.6, 139.9, 132.9, 130.1, 128.9, 128.5, 128.37, 125.7, 119.7, 118.1, 112.8, 53.4.

1-(2-methoxybenzyl)-4-phenyl-1H-1,2,3-triazole (entry 7)

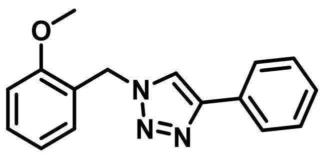

Chemical Formula: $C_{16}H_{15}N_3O$ White crystalline solid, 37 mg (28%).

1H NMR (400 MHz, Chloroform-d): δ 7.83 (d, J = 7.5 Hz, 2H), 7.74 (s, 1H), 7.51 – 7.19 (m, 6H), 7.06 – 6.87 (m, 2H), 5.62 (s, 2H), 3.91 (s, 3H).

13C{H}- NMR (101 MHz, Chloroform-*d*): δ. 157.1, 130.6, 130.3, 128.7, 128.0, 125.7, 122.9, 121.0, 110.8, 55.5, 49.2.

HRMS (ESI) m/z: [M + H]+ calcd. for $C_{16}H_{16}N_3O$ 266.1288; found: 266.1290

1-(cyclohexylmethyl)-4-phenyl-1H-1,2,3-triazole (entry 8)

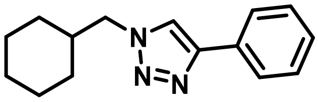

Chemical Formula: $C_{15}H_{19}N_3$ White amorphous solid, 70 mg (58%).

1H NMR (400 MHz, DMSO-d6): δ 8.56 (s, 1H), 7.93 – 7.76 (m, 2H), 7.45 (t, J = 7.6 Hz, 2H), 7.33 (t, J = 7.4 Hz, 1H), 4.25 (d, J = 7.1 Hz, 2H), 1.87 (m, 1H), 1.75 – 1.43 (m, 4H), 1.31 – 1.14 (m, 4H), 1.00 (m, 2H).

13C{H}- NMR (101 MHz, Chloroform-d): δ 147.5, 130.7, 128.8, 128.0, 119.9, 56.6, 38.8, 30.5, 26.0, 25.5.

HRMS (ESI) m/z: [M + H]+ calcd. for $C_{15}H_{20}N_3$ 242.1652; found: 242.1653

1-(2-cyclohexylethyl)-4-phenyl-1H-1,2,3-triazole (entry 9)

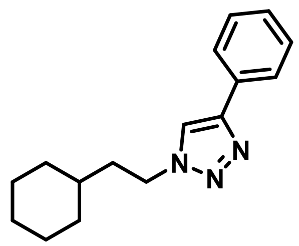

Chemical Formula: $C_{16}H_{21}N_3$ White amorphous solid, 111 mg (87%).

1H NMR (400 MHz, DMSO-d6): δ 8.46 (s, 1H), 7.72 (d, J = 7.1 Hz, 2H), 7.31 (t, J = 7.6 Hz, 2H), 7.19 (t, J = 7.4 Hz, 1H), 4.28 (t, J = 7.4 Hz, 2H -CH2-), 1.73 – 1.36 (m, 7H), 1.12 – 0.66 (m, 6H).

13C{H}- NMR (101 MHz, Chloroform-d): δ 147.7, 130.7, 128.8, 128.0, 125.6, 119.3, 48.2, 37.7, 34.9, 32.9, 26.3, 26.0.

HRMS (ESI) m/z: [M + H]+ calcd. for $C_{16}H_{22}N_3$ 256.1808; found: 256.1809

1-(5-(hydroxymethyl)-4-(4-phenyl-1H-1,2,3-triazol-1-yl)-2,5-dihydrofuran-2-yl)-5-methylpyrimidine-2,4(1H,3H)-dione (entry 10)

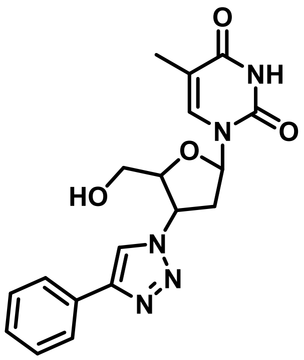

Chemical Formula: $C_{18}H_{19}N_5O_4$ White amorphous solid, 146 mg (79%).

1H NMR (400 MHz, DMSO-d6): δ 11.38 (s, 1H), 8.79 (s, 1H), 7.94 (d, J=8.2 Hz, 2H), 7.85 (s, 1H), 7.47 (t, J = 7.6 Hz, 2H), 7.41 – 7.24 (m, 1H), 6.46 (t, J = 6.6 Hz, 1H), 5.42 (dt, J = 8.7, 5.4 Hz, 1H), 5.31 (t, J = 5.2 Hz, 1H), 4.30 (dt, J = 5.5, 3.6 Hz, 1H), 3.80 – 3.55 (m, 2H), 2.88 – 2.62 (m, 2H), 1.83 (s, 3H).

13C{H}- NMR (101 MHz, DMSO): δ 164.2, 150.9, 147.0, 136.7, 131.0, 129.4, 128.4, 125.6, 121.4, 110.1, 84.9, 84.3, 61.2, 59.8, 37.6, 15.6, 12.7.

HRMS (ESI) m/z: [M + Na]+ calcd. for $C_{18}H_{19}N_5O_4$ Na 392.1329; found: 392.1329

2-(4-phenyl-1H-1,2,3-triazol-1-yl)tetrahydro-2H-pyran-3,4,5-triyl triacetate (entry 11)

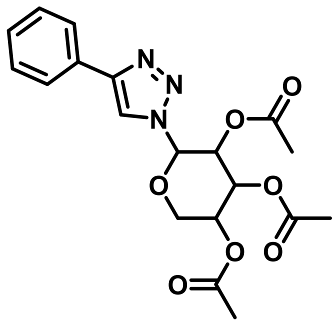

Chemical Formula: $C_{19}H_{21}N_3O_7$ White amorphous solid, 115 mg (57%).

1H NMR (400 MHz, Chloroform-d): δ 7.90 (s, 1H), 7.76 (d, J = 7.3 Hz, 2H), 7.37 (t, J = 7.5 Hz, 2H), 7.29 (t, J = 7.3 Hz, 1H), 5.80 – 5.75 (m, 1H), 5.43 – 5.34 (m, 2H), 5.12 (dq, J = 9.7, 5.5 Hz, 1H), 4.26 (dd, J = 11.6, 5.7 Hz, 1H), 3.60 – 3.52 (m, 1H), 2.02 (s, 3H), 2.00 (s, 3H). 1.83 (s, 3H).

13C{H}- NMR (101 MHz, Chloroform-d): δ 169.9, 169.8, 169.1, 148.4, 130.2, 129.9, 129.1, 128.9, 128.5, 125.9, 117.6, 86.4, 72.1, 70.3, 68.4, 65.6, 20.6, 20.6, 20.2.

HRMS (ESI) m/z: [M + Na]+ calcd. for $C_{19}H_{21}N_3O_7Na$ 426.1272; found: 426.1273

NMR spectra

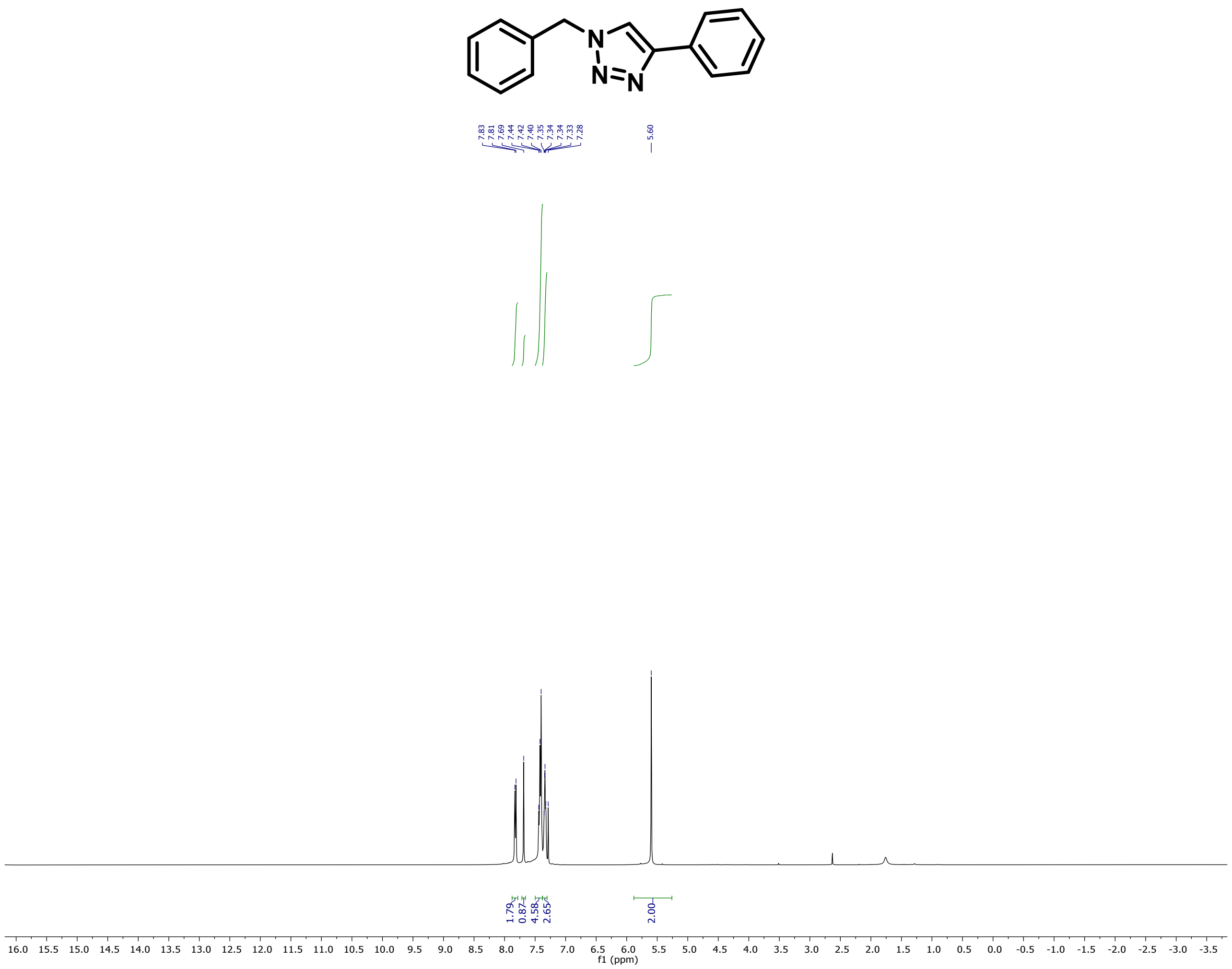

^1H NMR spectrum of compound 1 (400 MHz, $MeOD_4$)

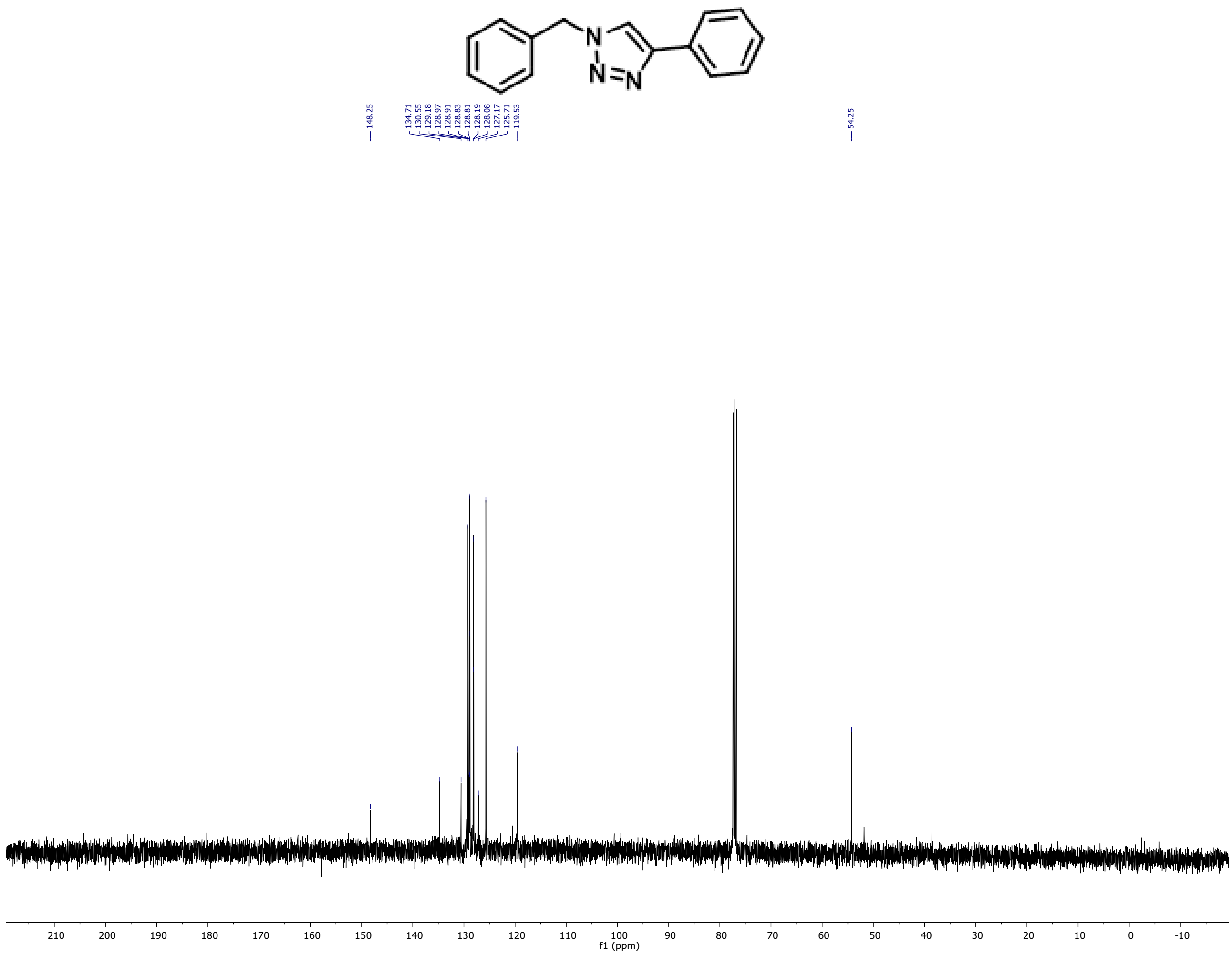

$^{13}C\{H\}$- NMR spectrum of compound 1 (101 MHz, $MeOD_4$)

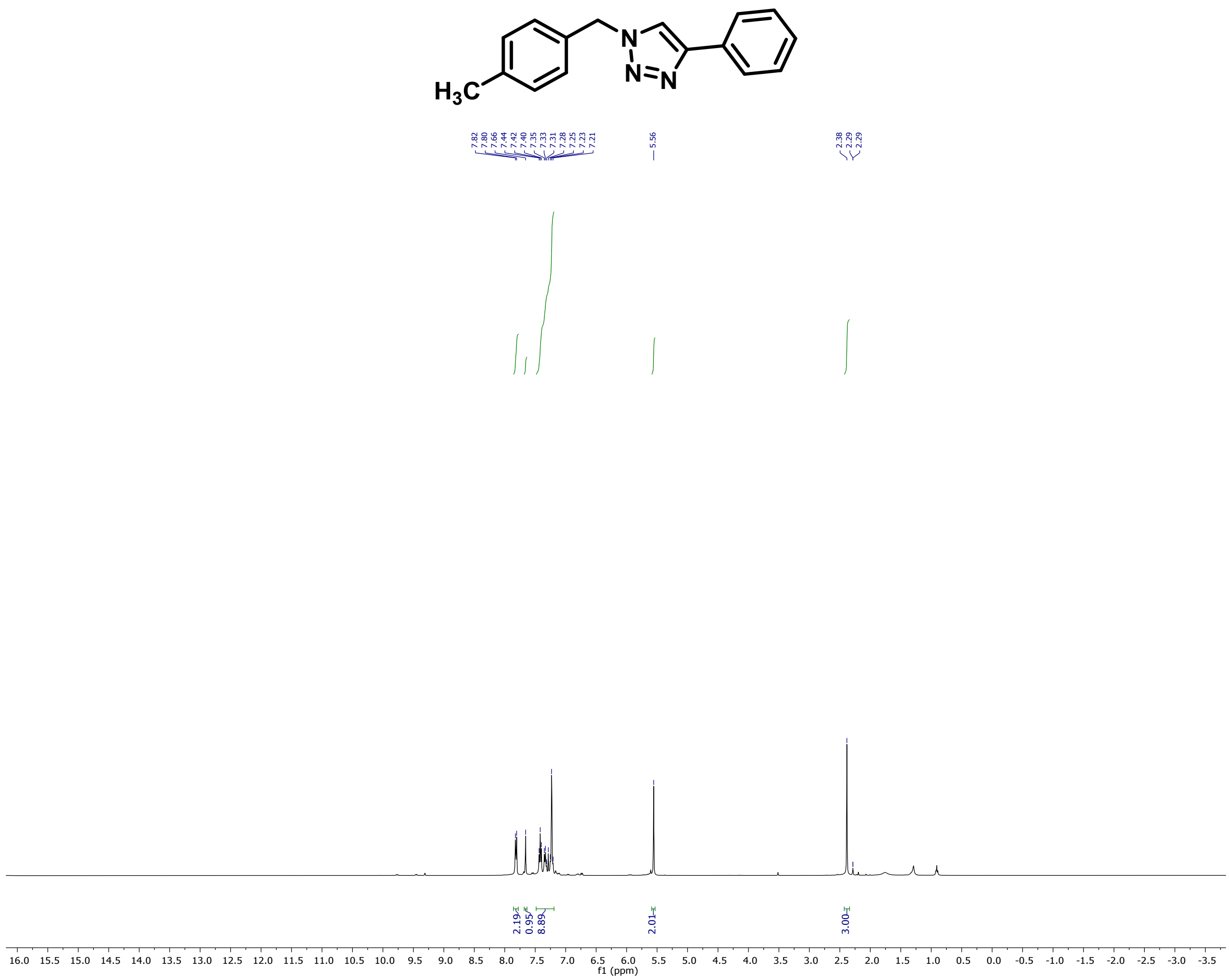

^{1}H NMR spectrum of compound 2 (400 MHz, Chloroform-d)

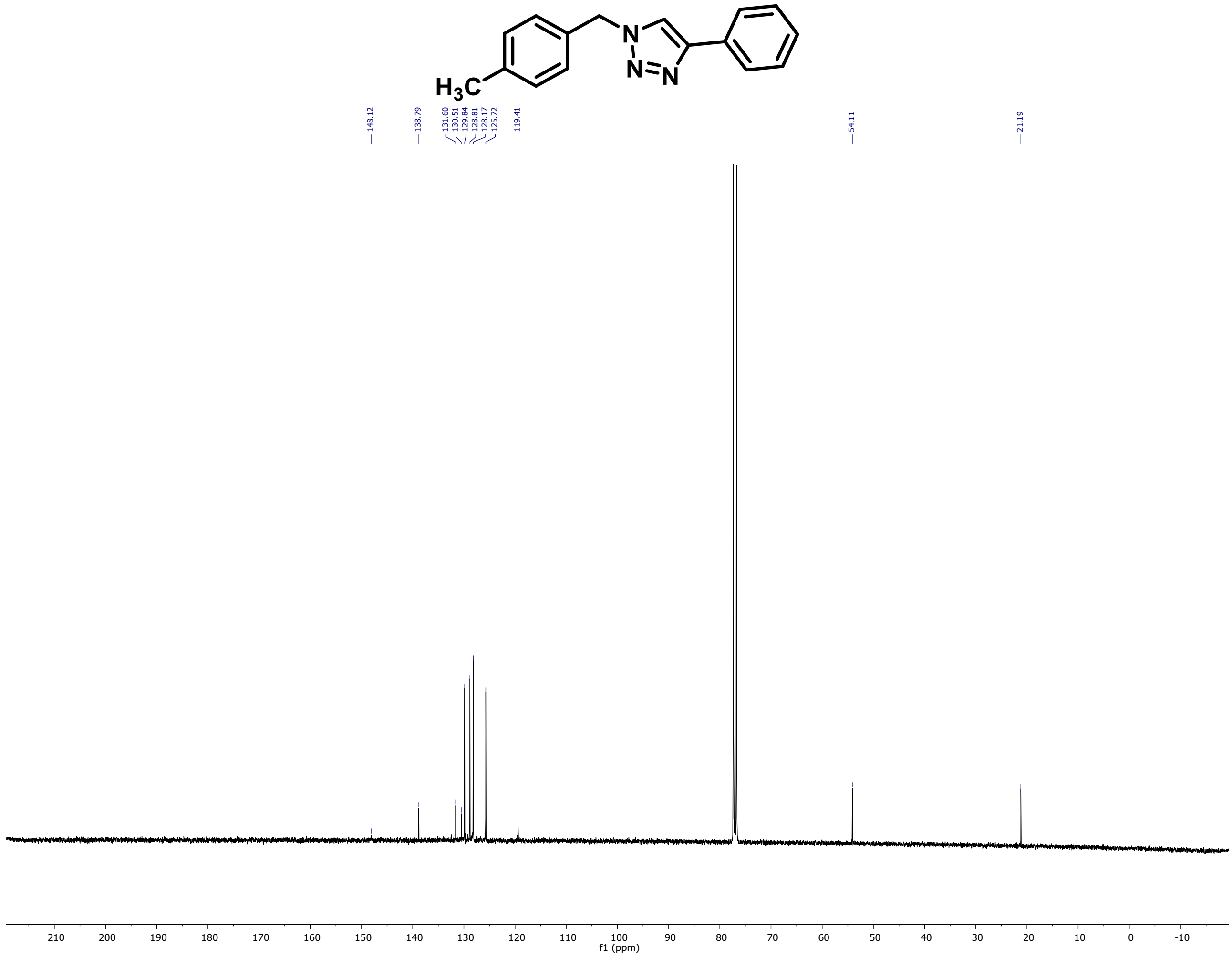

^{13}C{H}- NMR spectrum of compound 2 (101 MHz, Chloroform-d)

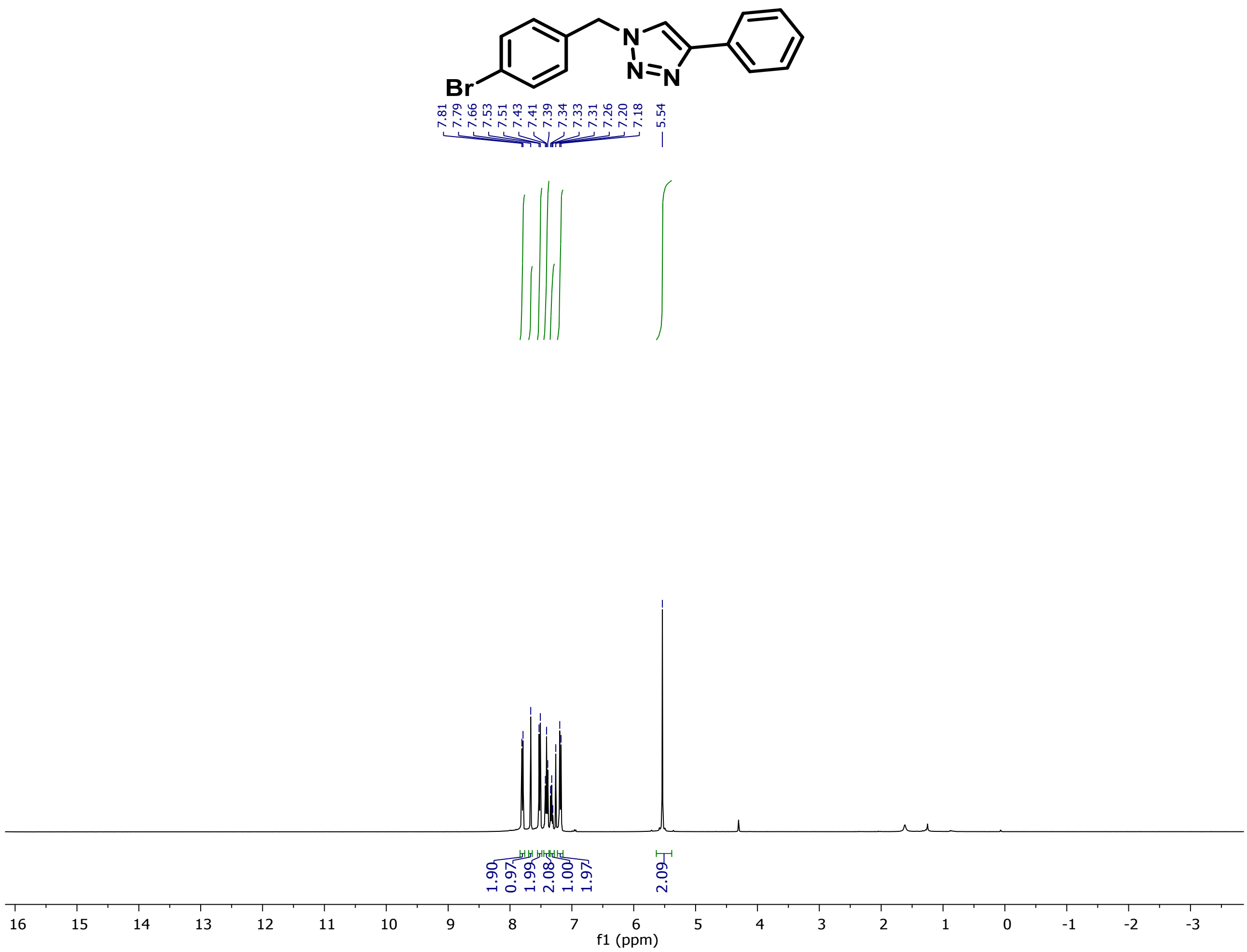

¹H NMR spectrum of compound 3 (400 MHz, Chloroform-d)

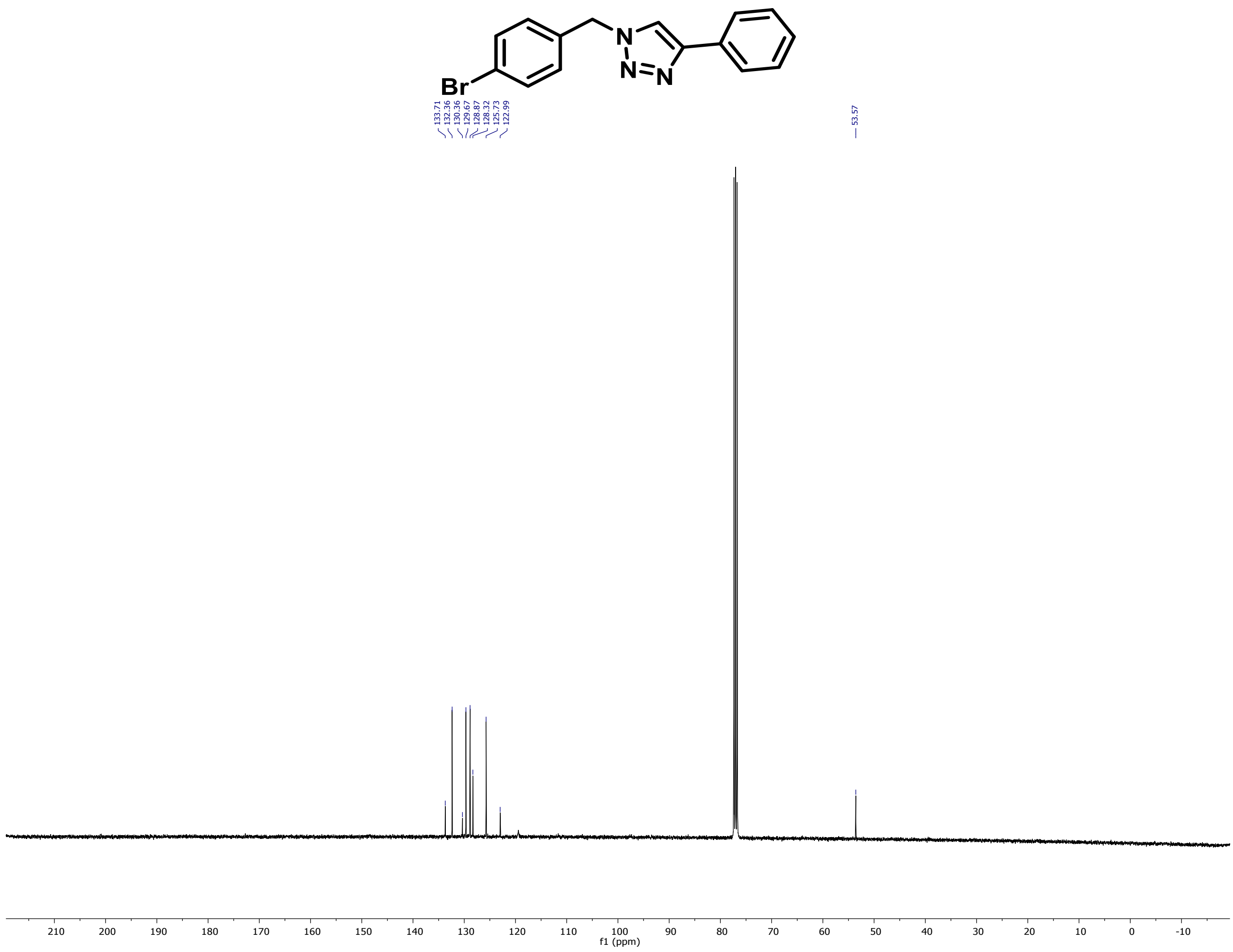

$^{13}C\{H\}$- NMR of compound 3 (101 MHz, Chloroform-d)

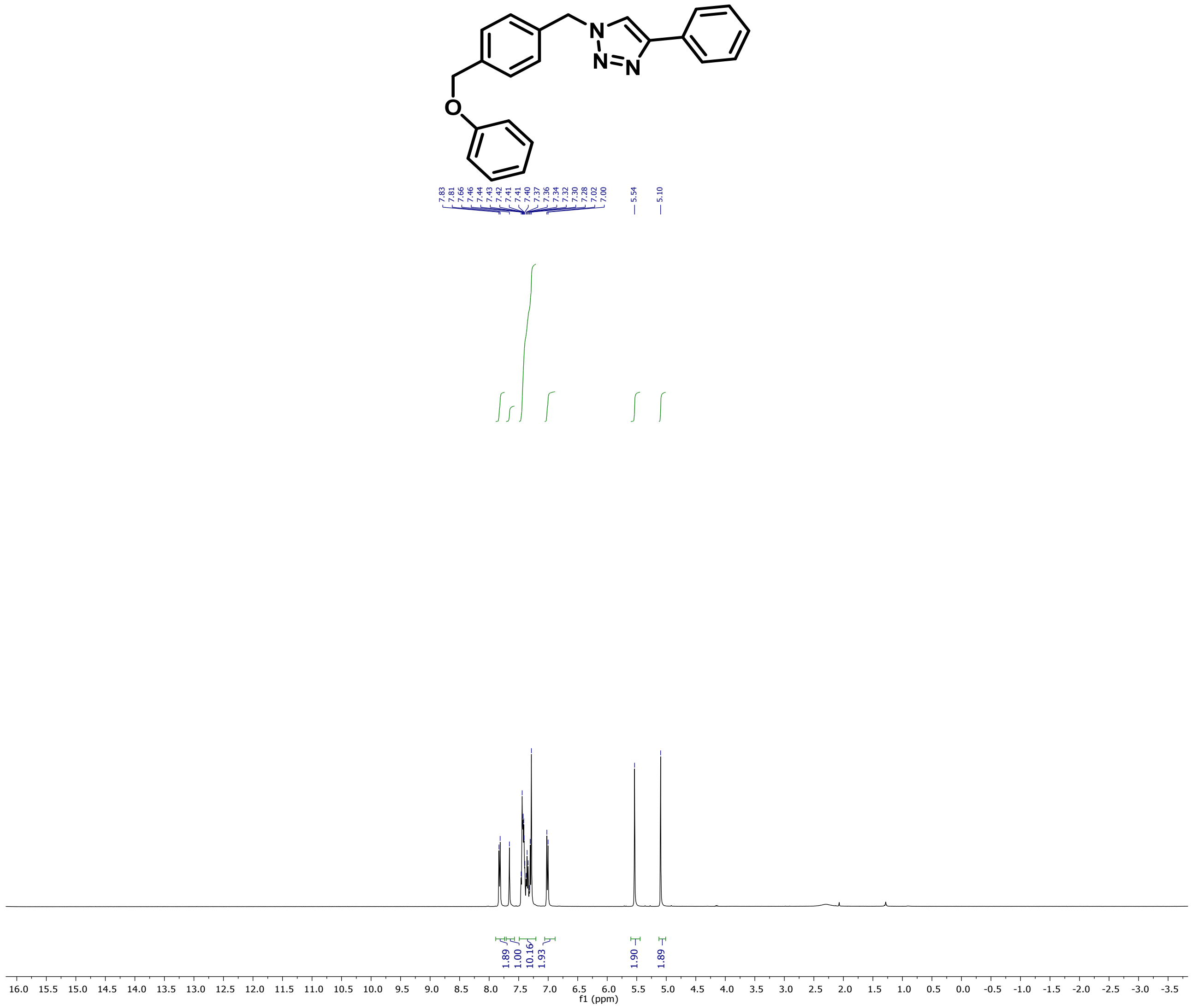

^{1}H NMR spectrum of compound 4 (400 MHz, Chloroform-d)

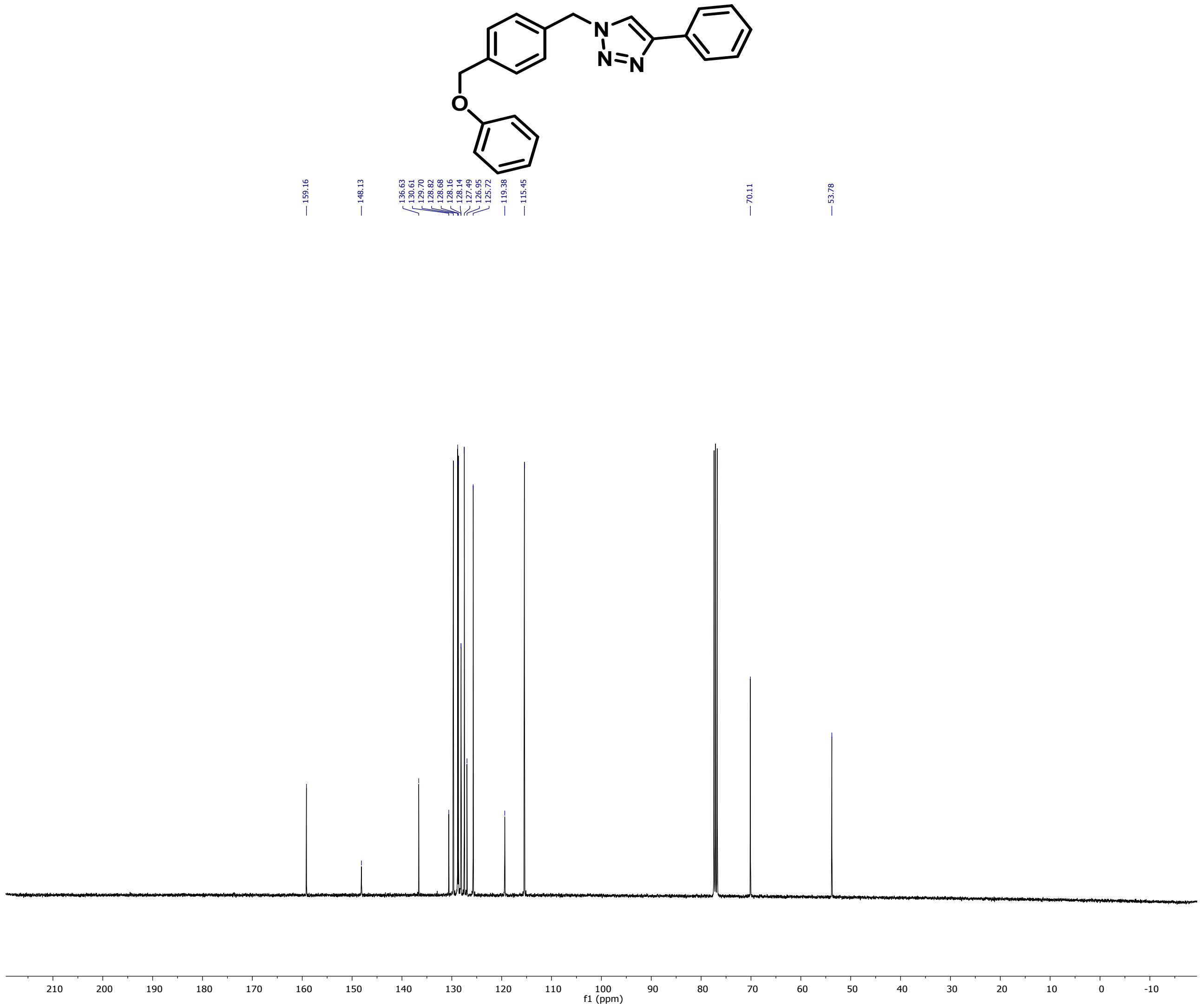

$^{13}C\{H\}$- NMR spectrum of compound 4 (101 MHz, Chloroform-d)

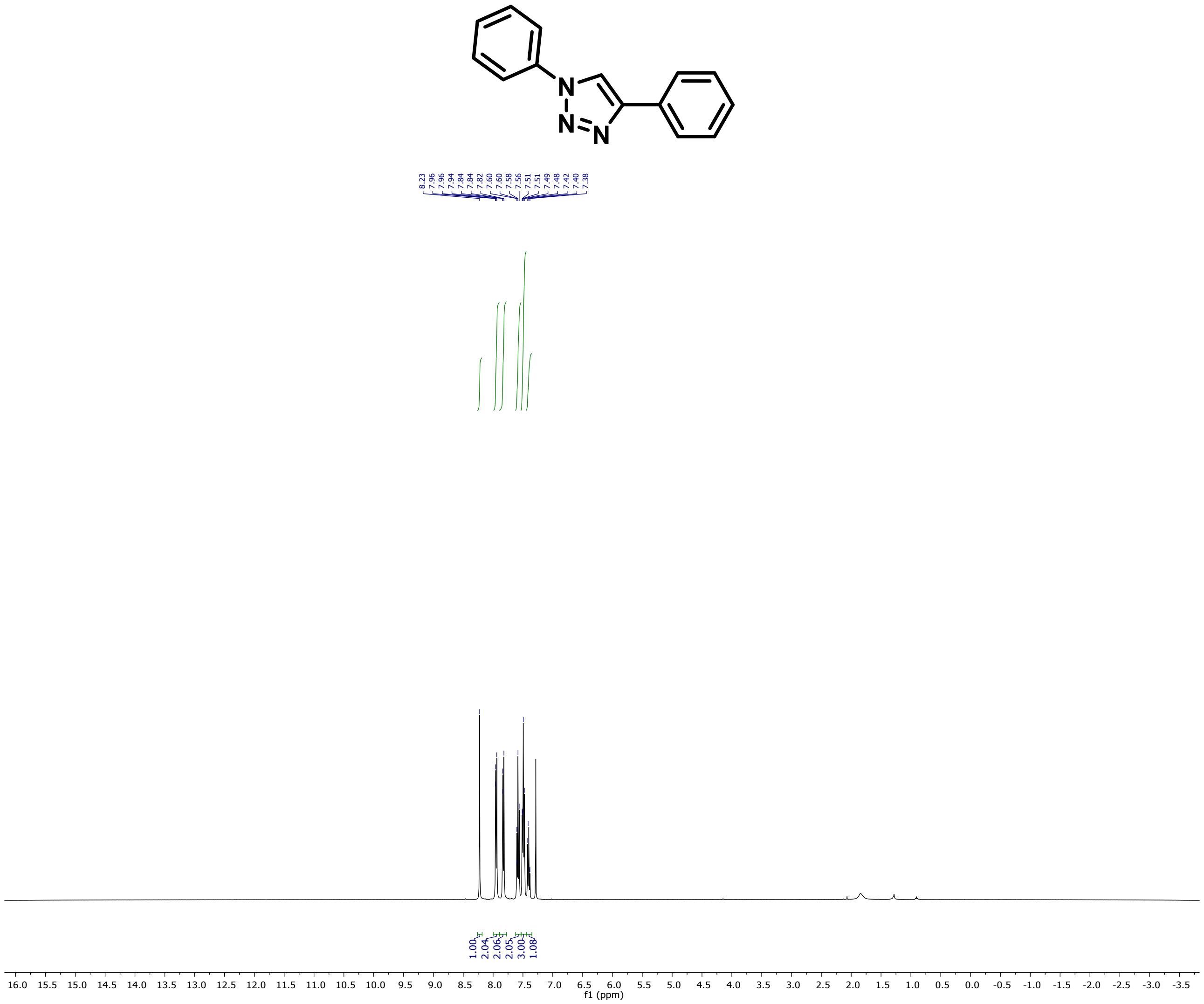

^{1}H NMR spectrum of compound 5 (400 MHz, Chloroform-d)

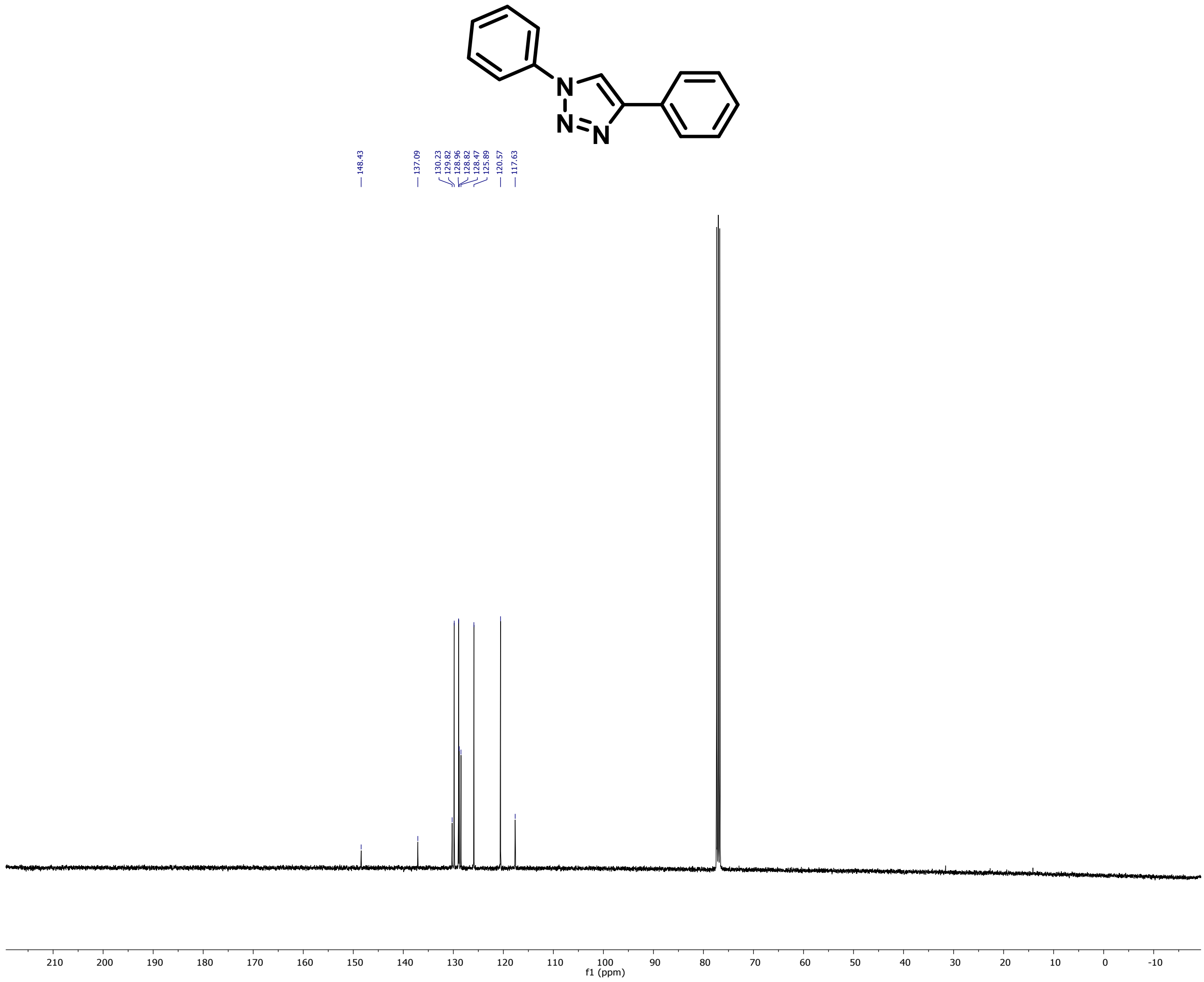

$^{13}C\{H\}$- NMR spectrum of compound 5 (101 MHz, Chloroform-d)

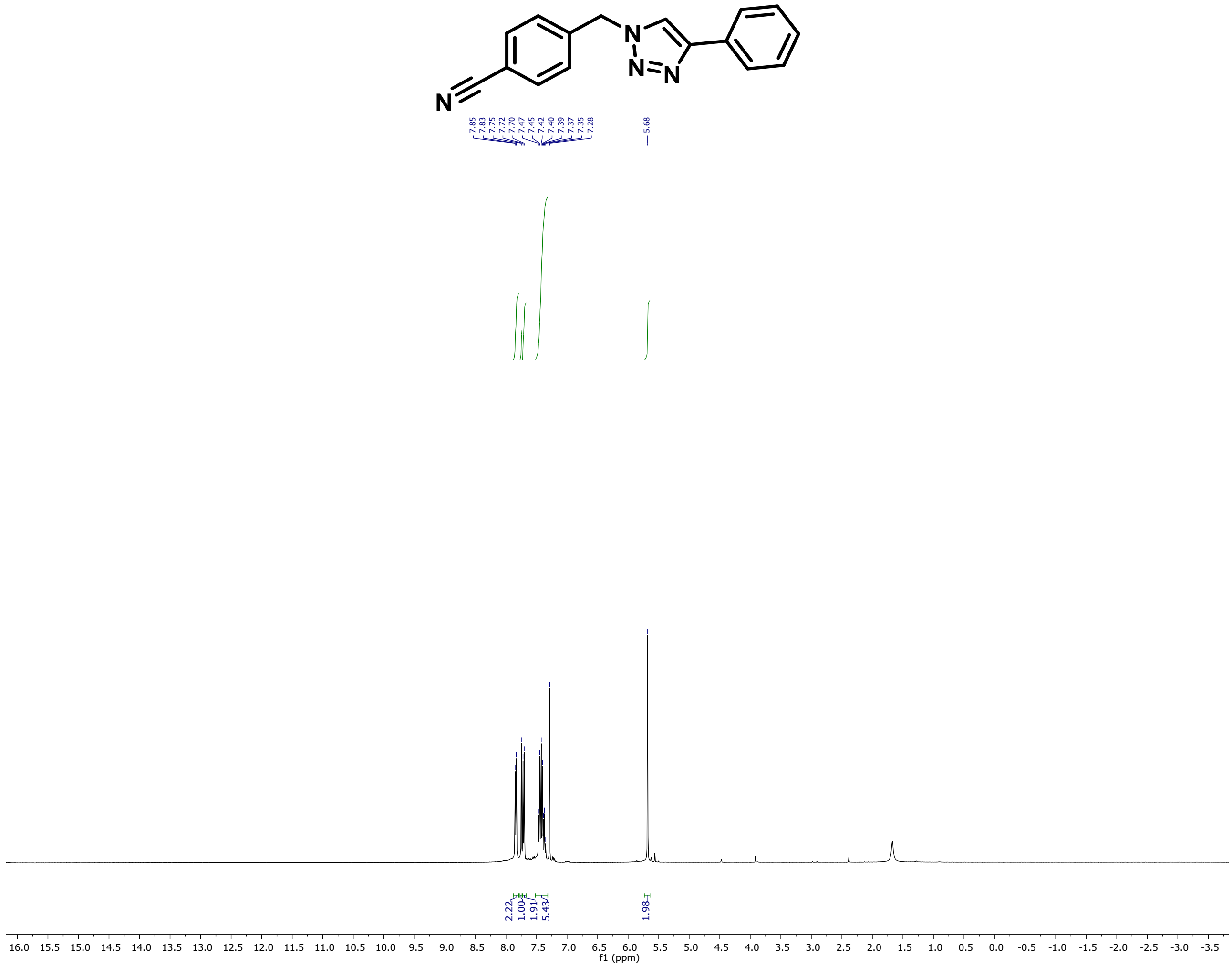

^{1}H NMR spectrum of compound 6 (400 MHz, Chloroform-d)

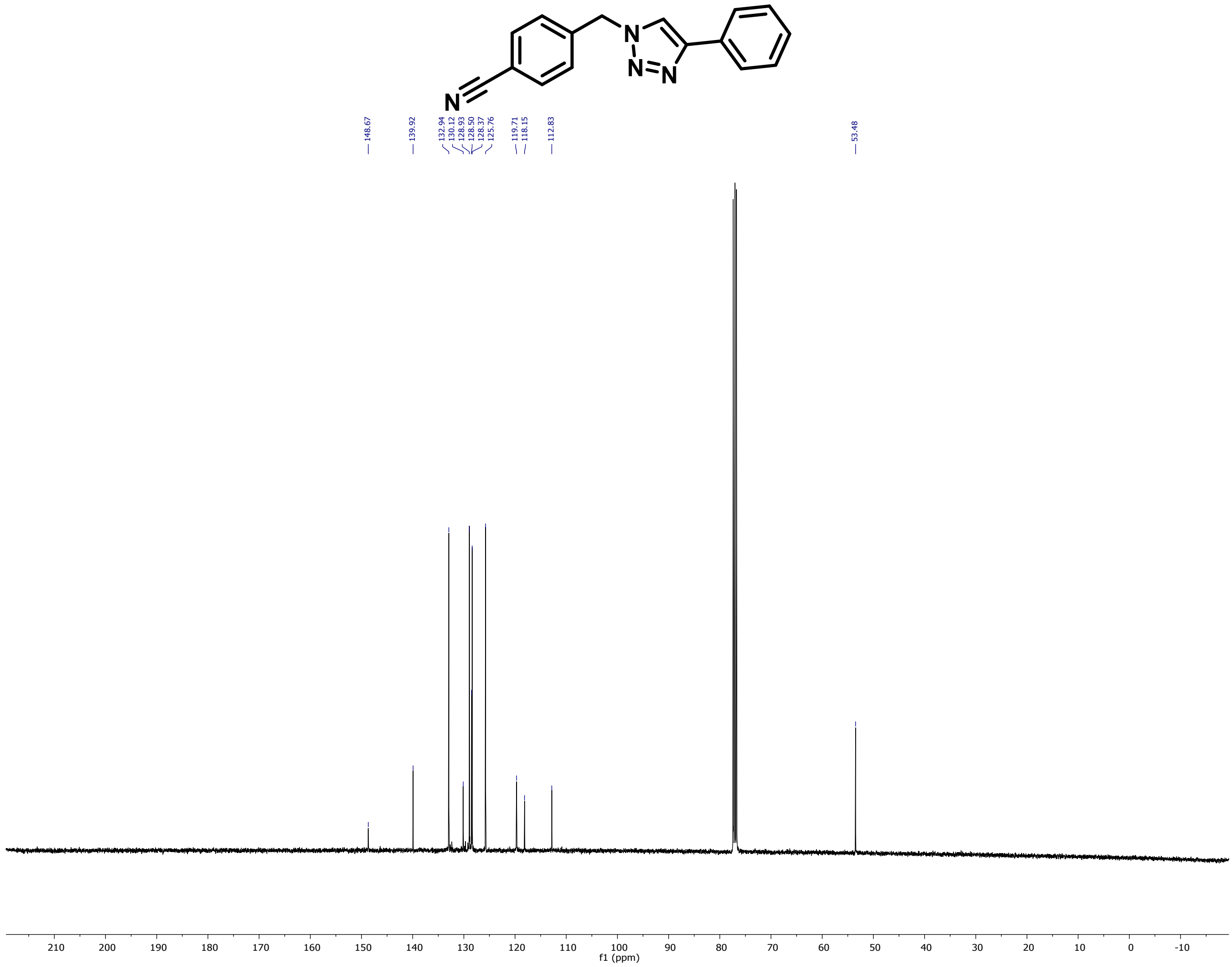

$^{13}C\{H\}$- NMR spectrum of compound 6 (101 MHz, Chloroform-d)

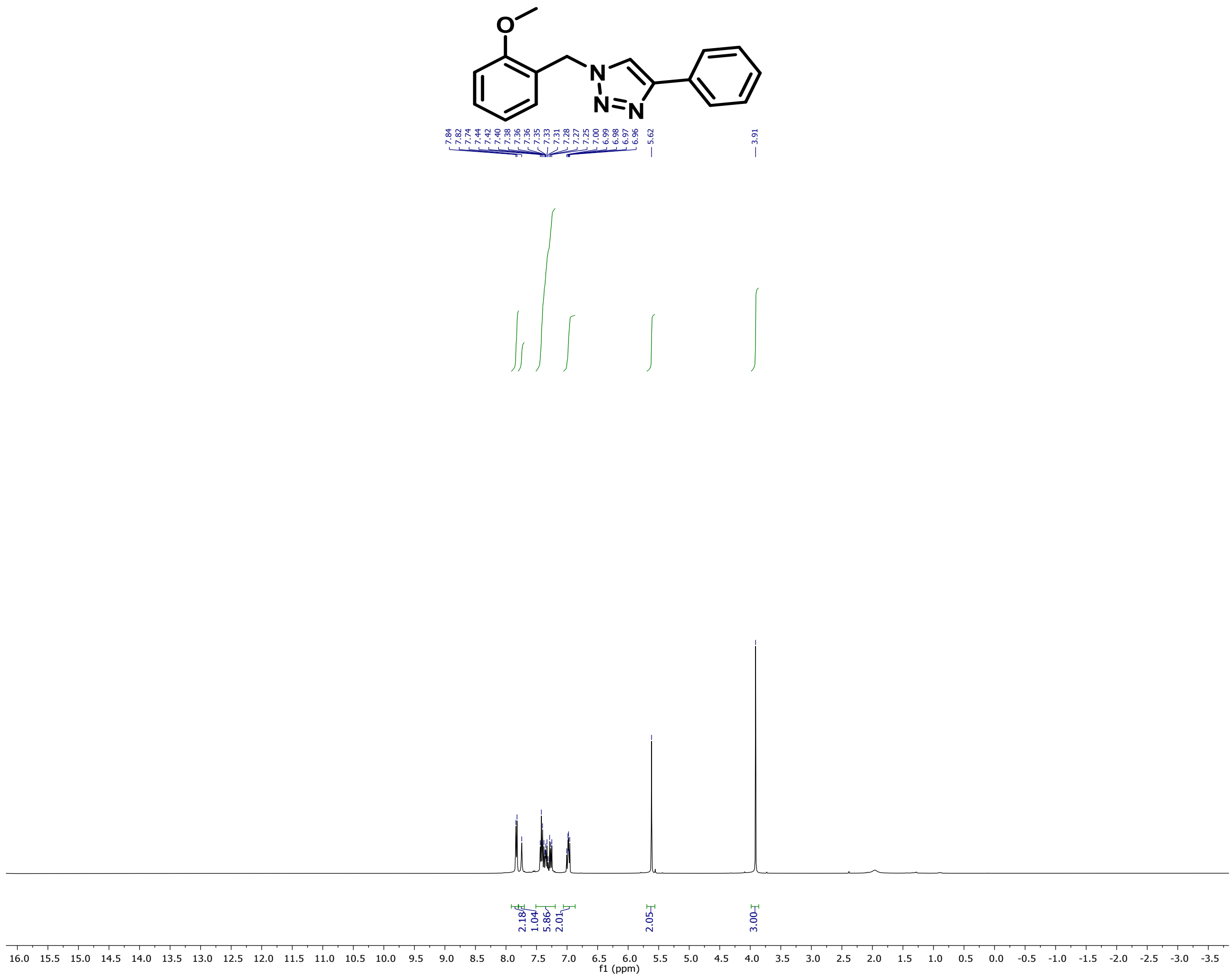

^{1}H NMR spectrum of compound 7 (400 MHz, Chloroform-d)

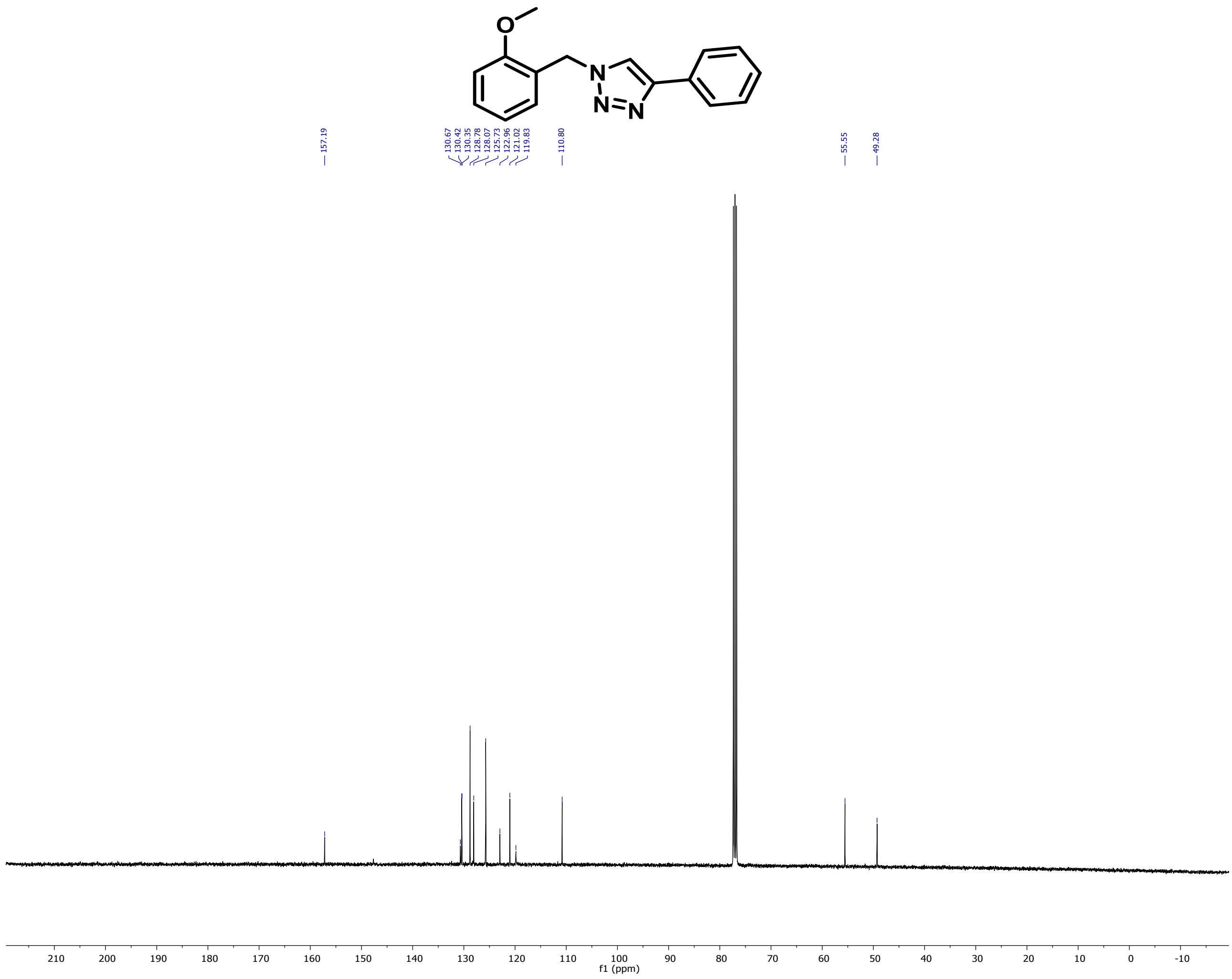

$^{13}C\{H\}$- NMR spectrum of compound 7 (101 MHz, Chloroform-d)

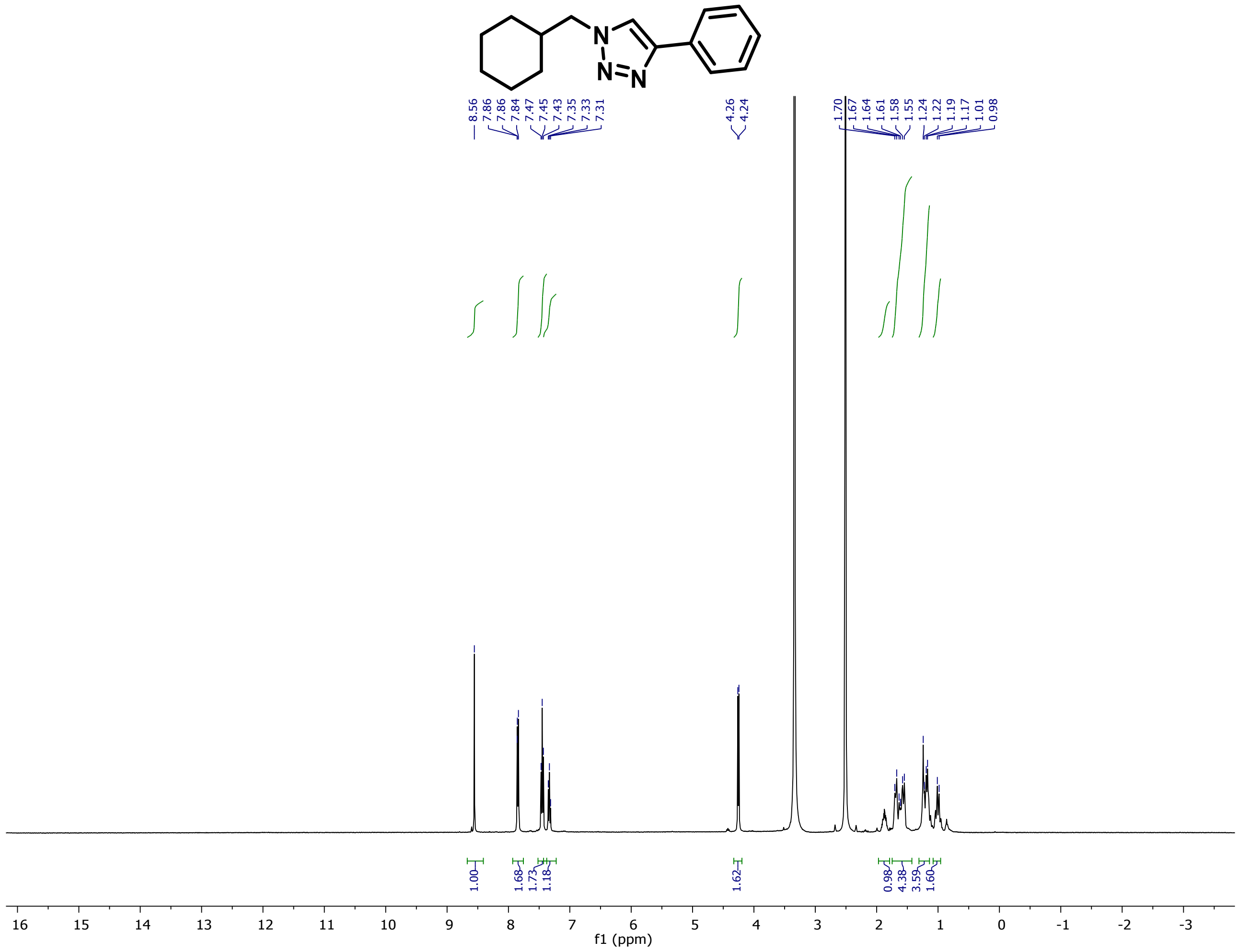

^{1}H NMR spectrum of compound 8 (400 MHz, DMSO-d_6)

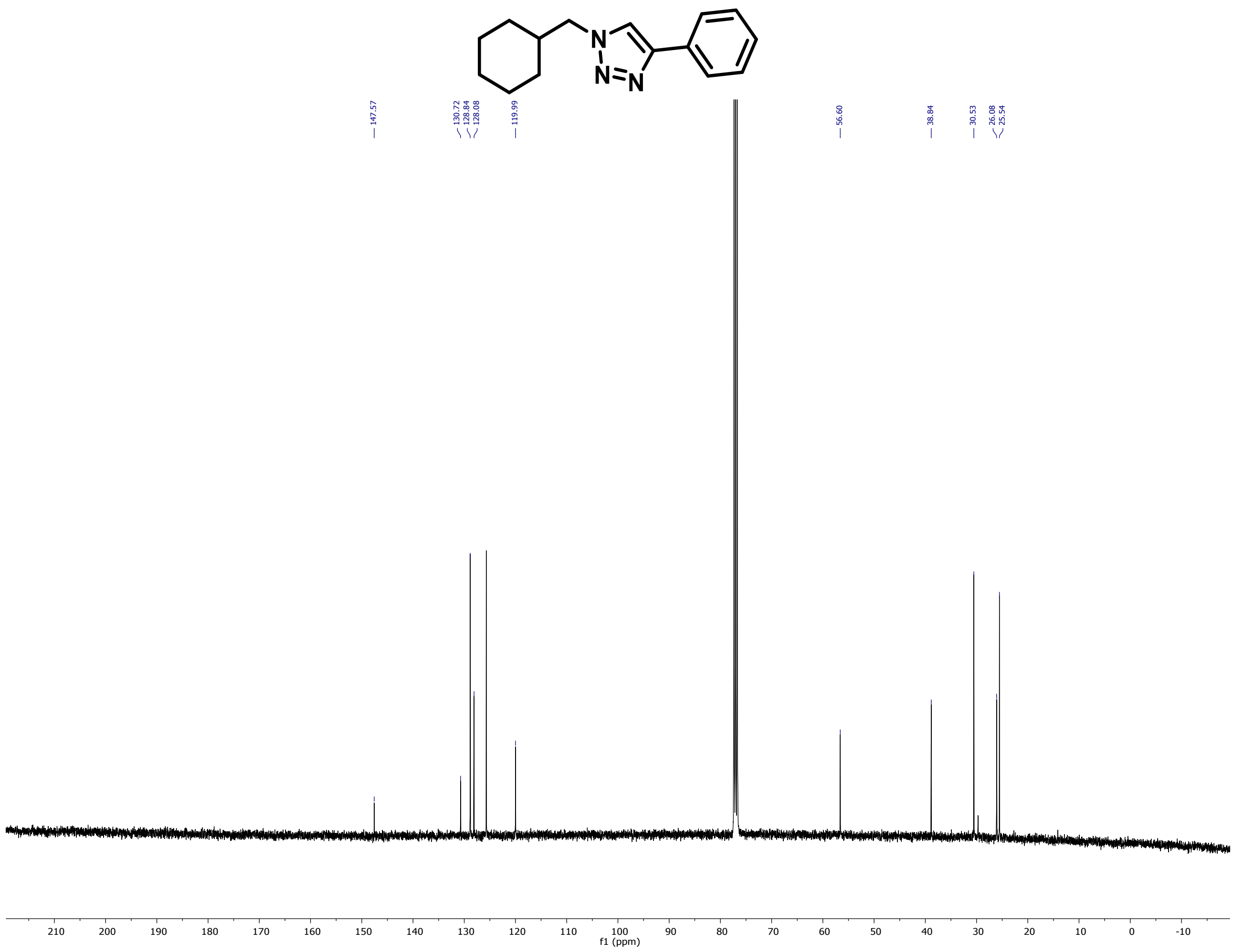

$^{13}C\{H\}$- NMR spectrum of compound 8 (101 MHz, Chloroform-d)

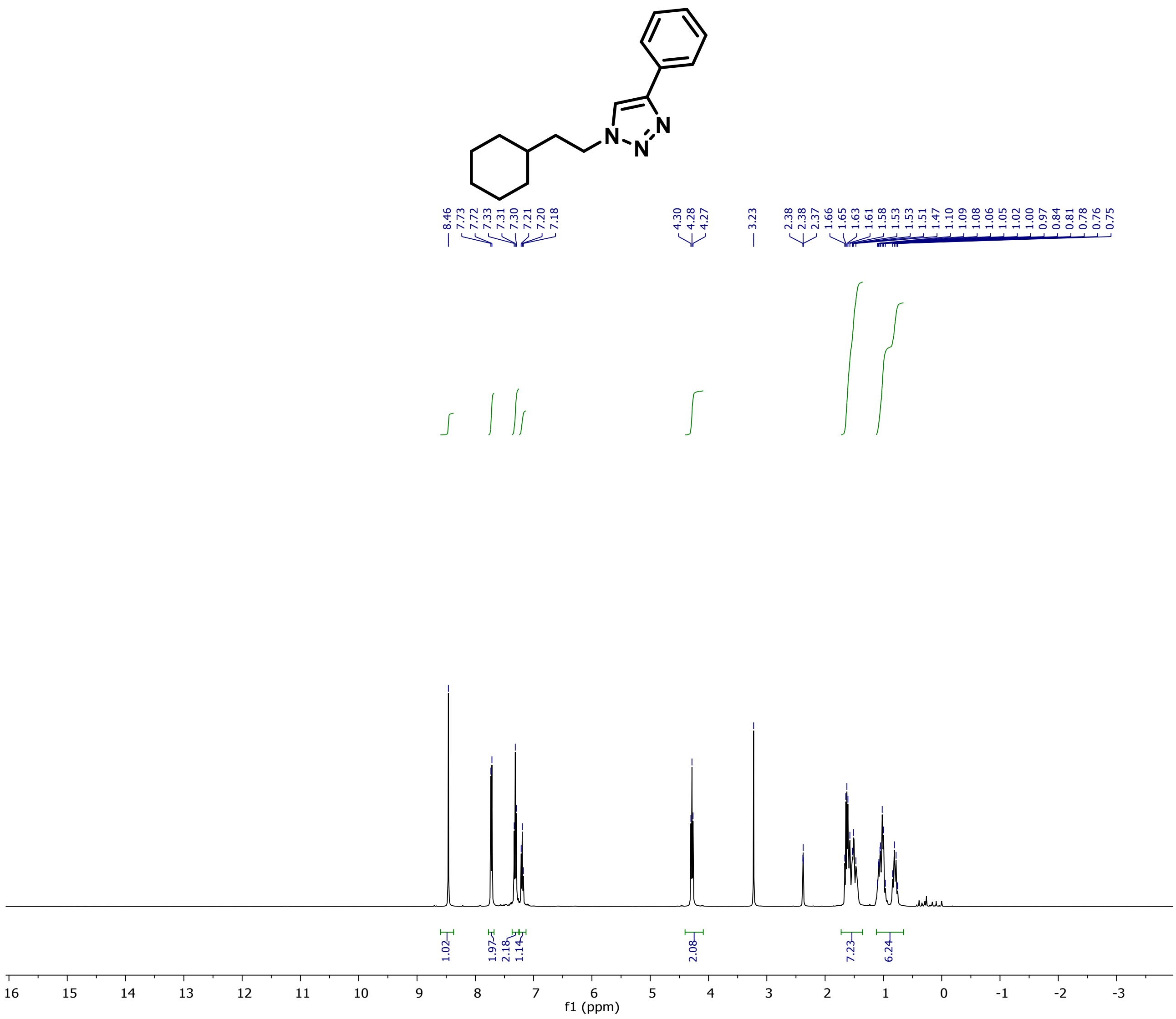

^{1}H NMR spectrum of compound 9 (400 MHz, DMSO-d_6)

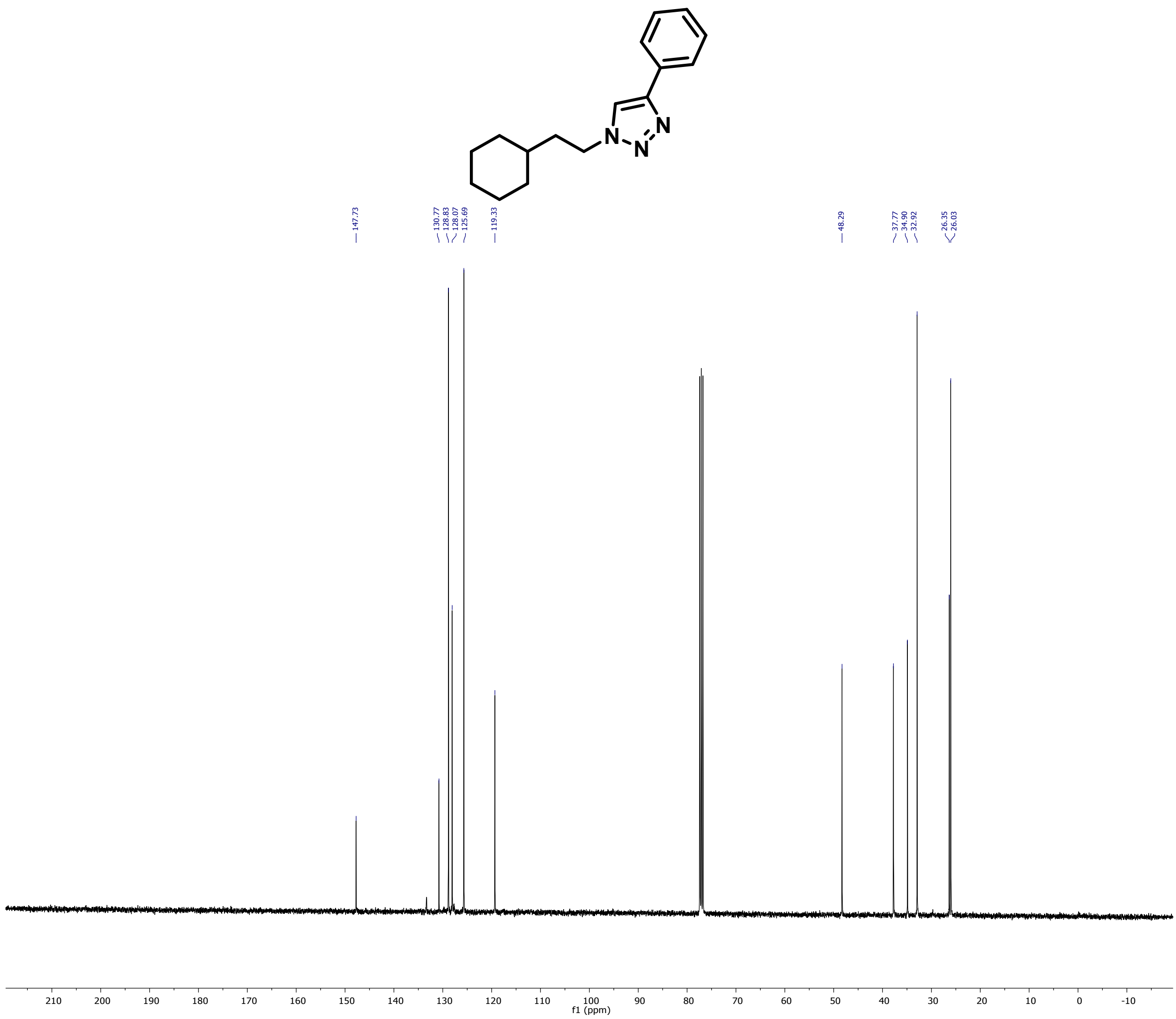

^{13}C{H}- NMR spectrum of compound 9 (101 MHz, Chloroform-*d*)

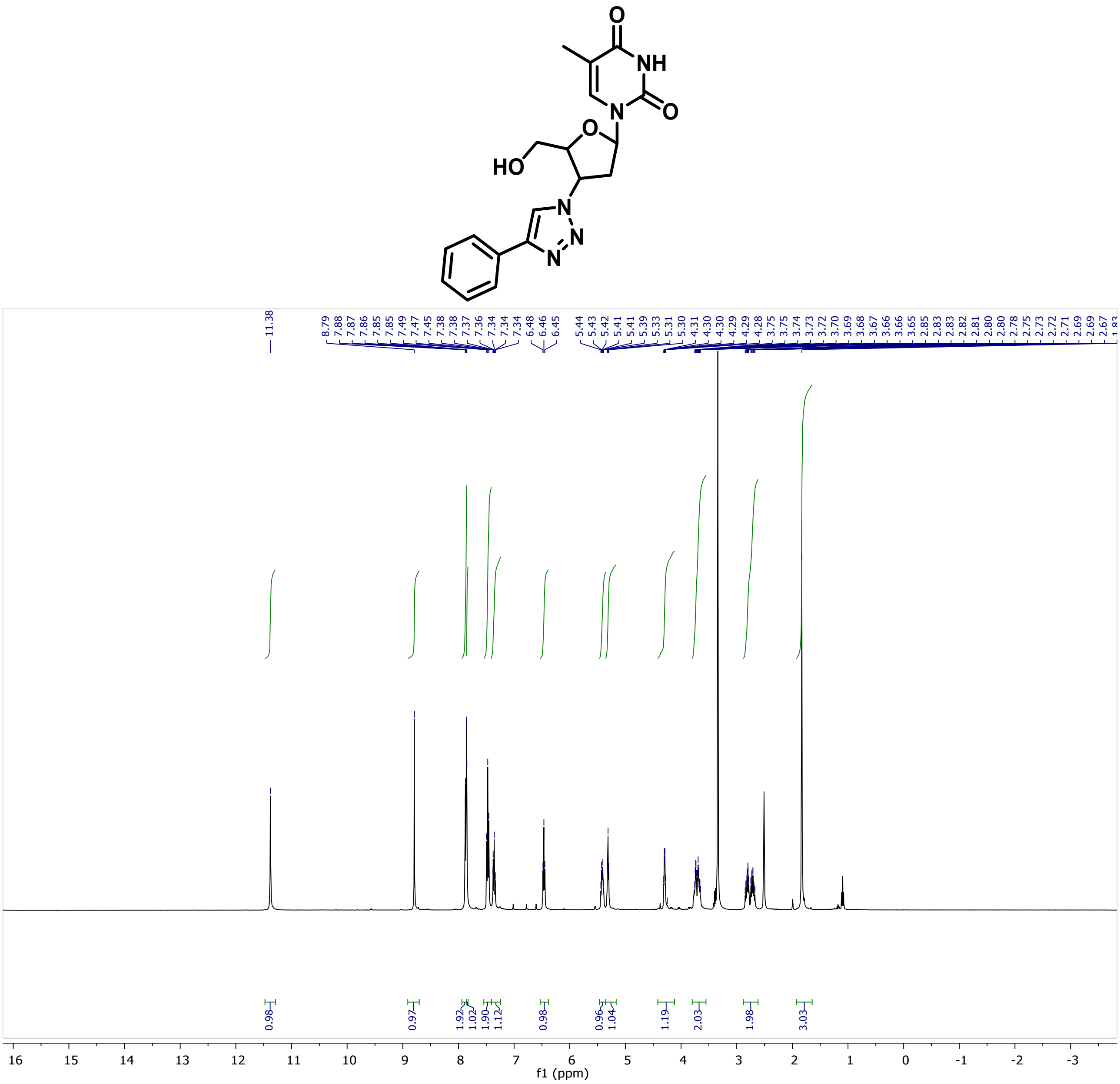

^{1}H NMR spectrum of compound 10 (400 MHz, DMSO-d6)

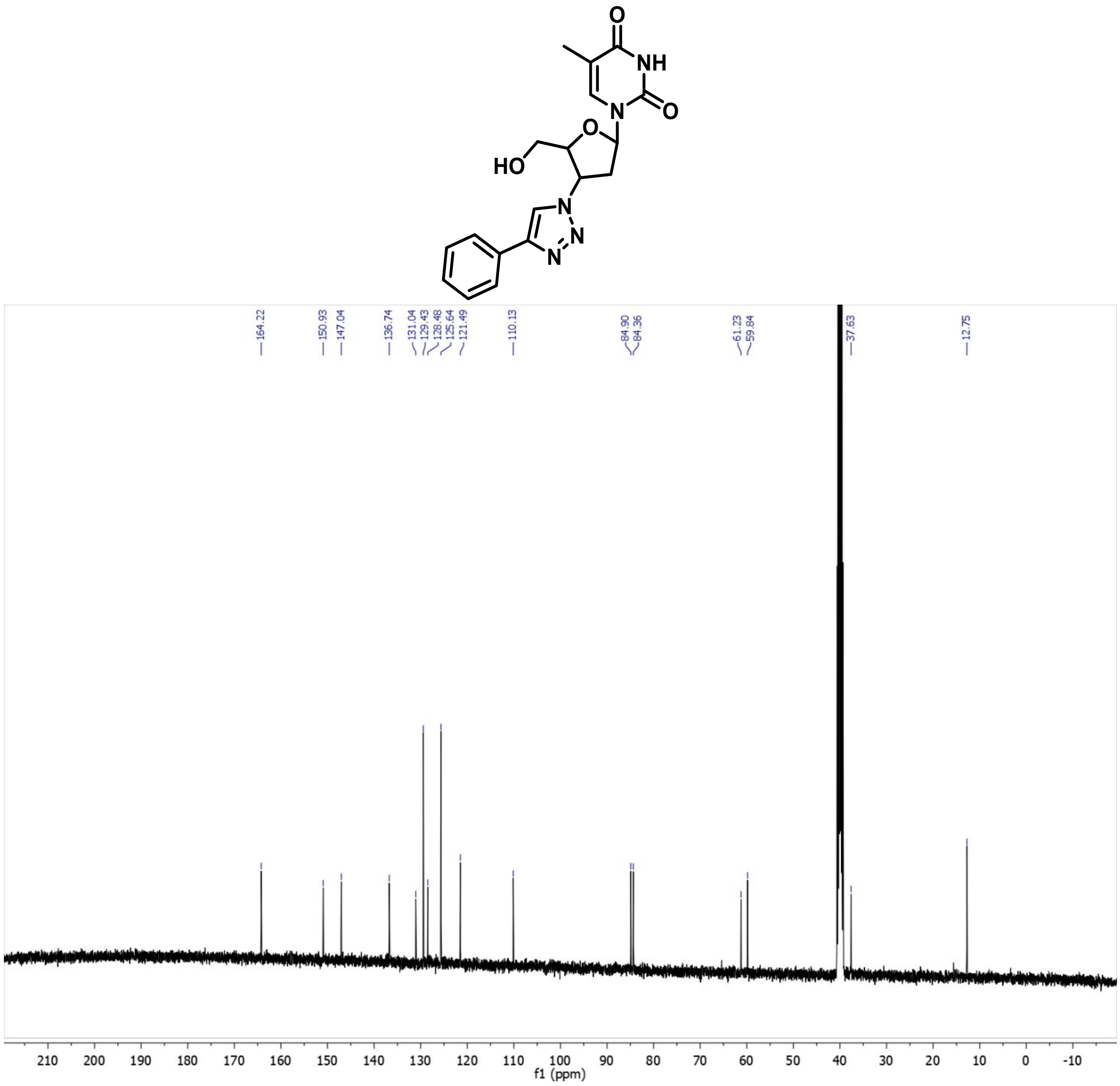

$^{13}C\{H\}$- NMR spectrum of compound 10 (101 MHz, DMSO-d6)

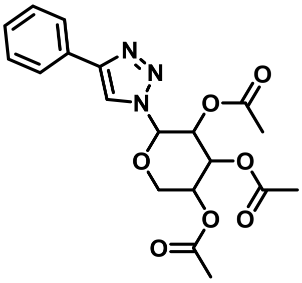

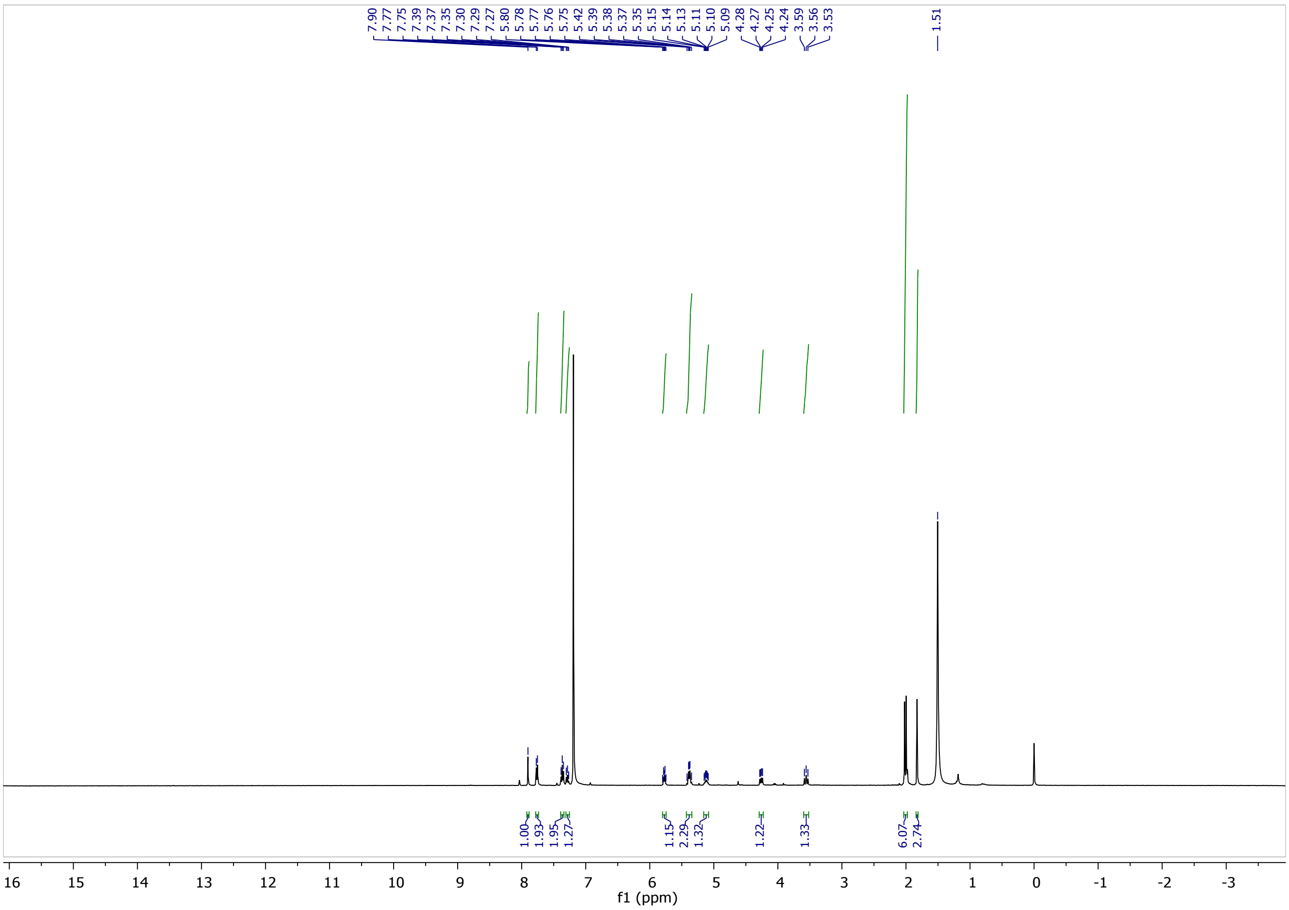

^{1}H NMR spectrum of compound 11 (400 MHz, Chloroform-d)

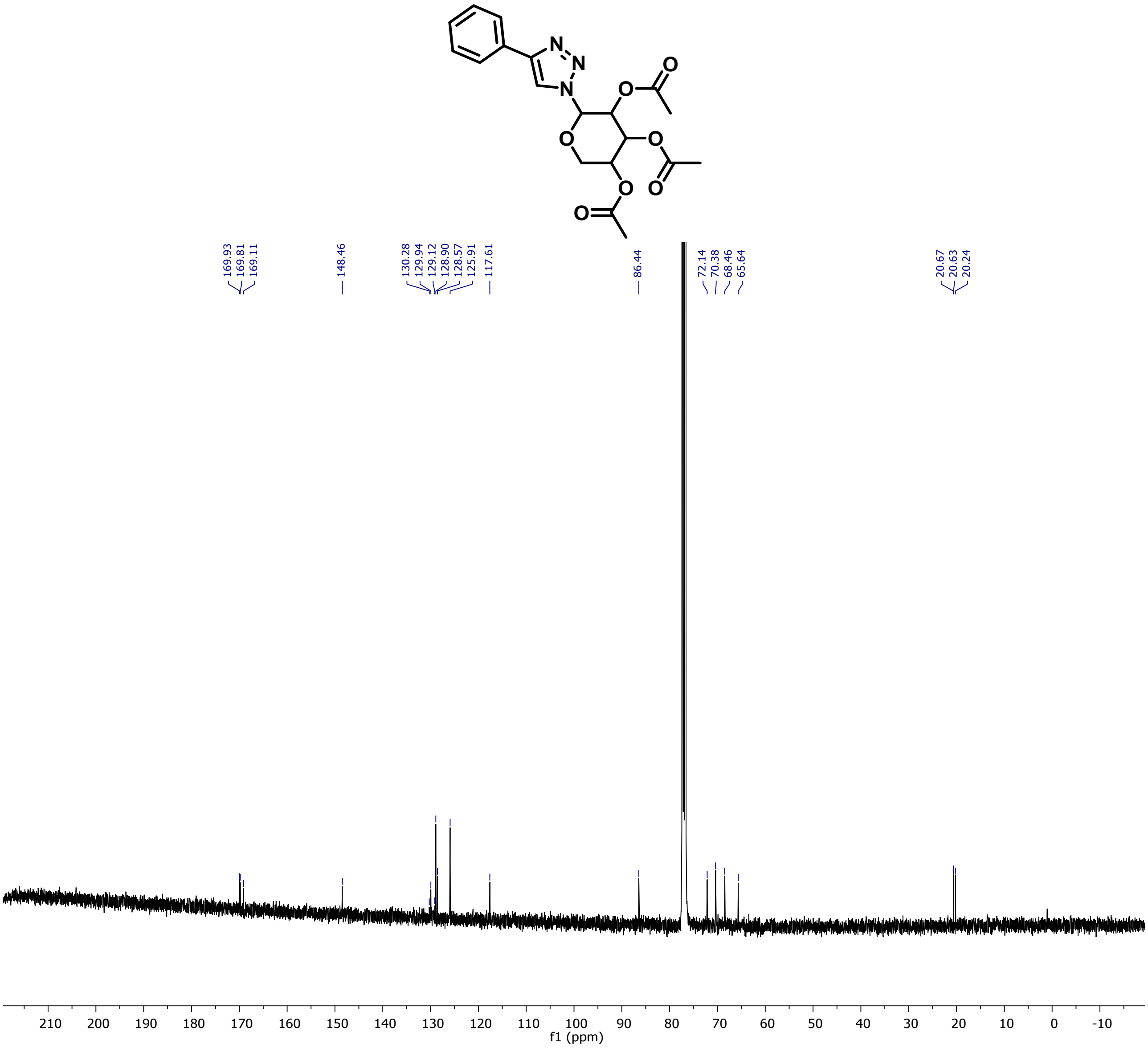

13C{H}- NMR spectrum of compound 11 (101 MHz, Chloroform-d)

pXRD spectra after each catalaytic cycle

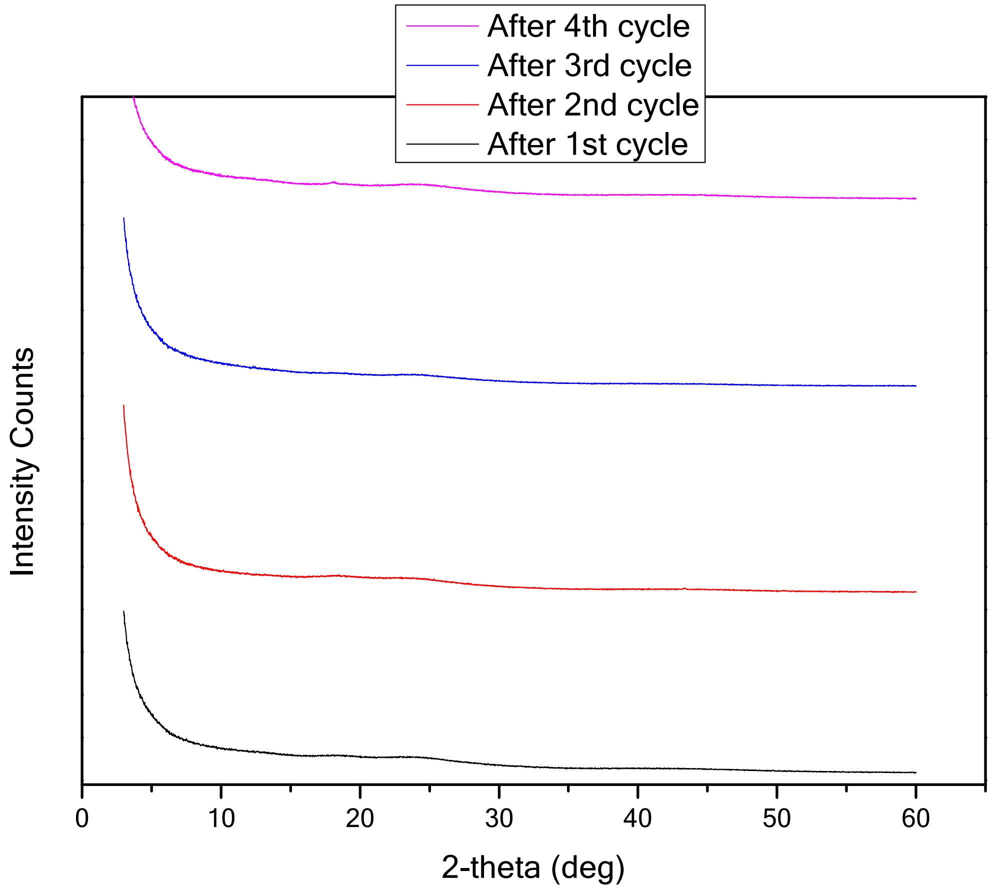

Inductively Coupled Plasma Optical Emission Spectroscopy (ICP-OES)

The analyses were performed using iCAP 7200 ICP-OES Duo from Thermo Fisher Scientific, USA, in dual view (radial and axial configuration). Acid digestion of the samples has been performed using Ethos UP Microwave Digestion System from Milestone, Italy, equipped with PTFE-TFM SK-15 easyTEMP vessels from Milestone. Samples were transferred into the ICP-OES with the autosampler ASX-560 from Teledyne Cetac Technologies, USA.

Standard solutions were prepared using the Certipur® ICP multi-element standard solution XVI (from Sigma-Aldrich) at 100 mg/L in nitric acid. Suprapur® Hydrochloric acid 36% and Suprapur® Nitric acid 69% for the preparation and dilution of the solutions were obtained from Sigma-Aldrich. Ultrapure water (with a resistivity of 18.2 MΩ·cm) was obtained using the purification system Direct-Q® 3 UV Millipore, using Biopak® filter by Merck, Germany.
Argon gas 5.0, purity ≥ 99.999% mol, was obtained from Nippon Gases Rivoira.
Data were managed and analysed using the software Qtegra ISDS (Intelligent Scientific Data Solution Software) from Thermo Fisher Scientific.

Methods

Preliminary operations: First, calibration curves were obtained using solutions at different Cu concentrations: 0.1 ppb to 100 ppm. The samples were diluted using a solution with 1% V/V of hydrochloric acid and 1% V/V of nitric acid, prepared using the Suprapur concentrated acid solutions and ultrapure water. The same solution has been used as running and washing solution for the ICP-OES, as well as rinse solution for the autosampler. The emission wavelengths of copper have been chosen among the most intense ones and to avoid overlapping with the emission of other elements and have been reported in **Table SI 1**. The R^2 value for all calibration curves was ≥ 0.99.

Preparations of the samples: Approximately 300 mg of the samples were weighted, inserted in SK-15 vessels and diluted with 10 ml of inverse *aqua regia* (a mixture of hydrochloric acid and nitric acid in 1:3 ratio). The SK-15 vessels were then closed and inserted into the microwave digester digestion system. The digestion program consisted of a linear increase of the temperature up to 225°C for 25 minutes, another linear increase to 240°C for 30 min and then 30 minutes for the cooling of the vessels inside the instrument. After the digestion, the solutions were transferred into Falcon tubes and diluted to a final volume of 50 mL using ultrapure water. Samples were then sonicated for 15 minutes and centrifugated for 5 min (7830 rpm). No residues were observed on the bottom of the tubes. The solutions were ready for analysis. A blank solution was prepared following the same steps described above, with the only exception of the absence of the sample in the solution.

Analysis: The solutions were analysed both in radial and axial configuration with the instrumental parameters described in **Table SI 1**. Each analysis has been performed in triplicate and the results have been expressed as mean value ± standard deviation.

Table S1

Emission wavelengths	
**Cu**	324.754 nm (I); 224.700 nm (II)
**Ar**	404.442 nm (I)

Parameter	Setting	Parameter	Setting
**RF power**	1150 W	**Pump speed**	45 rpm
**Viewing mode**	Radial and axial	**Wash time**	30 s
**Nebulizer gas flow rate**	0.5 L/min	**Uptake time**	30 s
**Cooling gas flow rate**	12 L/min	**Number of replicates**	3
**Auxiliary gas flow rate**	0.5 L/min	**Plasma Gas Flow rate**	12 L/min
**Radial exposure time**	5 s (for UV/Vis)	**Flush pump speed**	45 rpm
**Axial exposure time**	5 s (for UV/Vis)		
